# Associations between Dengue Incidence, Ecological Factors, and Anthropogenic Factors in Singapore

**DOI:** 10.3390/v15091917

**Published:** 2023-09-13

**Authors:** Pranav Tewari, Peihong Guo, Borame Dickens, Pei Ma, Somya Bansal, Jue Tao Lim

**Affiliations:** 1 Lee Kong Chian School of Medicine, Nanyang Technological University, Singapore 308232, Singapore; pranav.tewari@ntu.edu.sg (P.T.); peihong.guo@ntu.edu.sg (P.G.); juetao.lim@ntu.edu.sg (J.T.L.); 2 Saw Swee Hock School of Public Health, National University of Singapore, Singapore 117549, Singapore; mapei@nus.edu.sg (P.M.); somya.b@nus.edu.sg (S.B.)

**Keywords:** dengue, non-linear associations, generalized additive models, Shapley values, Singapore, spatio-temporal analysis

## Abstract

Singapore experiences endemic dengue. Vector control remains the primary means to reduce transmission due to the lack of available therapeutics. Resource limitations mean that vector-control tools need to be optimized, which can be achieved by studying risk factors related to disease transmission. We developed a statistical modelling framework which can account for a high-resolution and high-dimensional set of covariates to delineate spatio-temporal characteristics that are associated with dengue transmission from 2014 to 2020 in Singapore. We applied the proposed framework to two distinct datasets, stratified based on the primary type of housing within each spatial unit. Generalized additive models reveal non-linear exposure responses between a large range of ecological and anthropogenic factors as well as dengue incidence rates. At values below their mean, lesser mean total daily rainfall (Incidence rate ratio (IRR): 3.75, 95% CI: 1.00–14.05, Mean: 4.40 mm), decreased mean windspeed (IRR: 3.65, 95% CI: 1.87–7.10, Mean: 4.53 km/h), and lower building heights (IRR: 2.62, 95% CI: 1.44–4.77, Mean: 6.5 m) displayed positive associations, while higher than average annual NO_2_ concentrations (IRR: 0.35, 95% CI: 0.18–0.66, Mean: 13.8 ppb) were estimated to be negatively associated with dengue incidence rates. Our study provides an understanding of associations between ecological and anthropogenic characteristics with dengue transmission. These findings help us understand high-risk areas of dengue transmission, and allows for land-use planning and formulation of vector control policies.

## 1. Introduction

Dengue virus (DENV) is responsible for the highest burden of disease among arboviruses, accounting for 100 million symptomatic infections [1] and 20,000 deaths annually across 195 countries [2]. Presently, nearly 50% of the global population resides in regions that are conducive for the transmission of DENV [3]. Dengue incidence has increased 30-fold over the past five decades, and projections estimate that 2.25 billion more people will be at risk of dengue within the next five decades. This upward trend in dengue incidence has been attributed to several drivers, including but not limited to: rapid unplanned urbanization [4], climate change [5], population growth [6], suboptimal water and solid waste management [7], and travel [8]. Globally, it is estimated that DENV incurs an annual economic burden of 2.1 billion USD [9]. According to the World Health Organization (WHO), Asia accounted for 75% of the worldwide dengue burden in 2012, with Southeast Asia alone incurring an annual cost of 1 billion USD [10].

The disease, which is caused by DENV transmission through the bites of infected female *Aedes* species mosquitos, can cause a wide range of symptoms ranging from mild fever to severe hemorrhagic fever and shock syndrome. There are no therapeutics available for dengue as of 2023. Current treatment approaches are supportive, focusing on mitigating complications and reducing the severity of symptoms [9]. A dengue vaccine, sold under the brand name Dengvaxia, is currently commercially available. However, its usage is constrained by its potential to heighten the risk of severe dengue in individuals with no prior infection history [11]. TAK-003, sold under the brand name QDenga, has demonstrated a promising safety profile [12]. While it has received marketing authorization valid throughout the EU [13], its efficacy has yet to be demonstrated in real-world applications. In the absence of therapeutics and widely available and effective vaccines, vector control remains the primary tool to mitigate dengue transmission [14]. The prevention and control of DENV therefore poses a daunting public health challenge in both tropical and subtropical regions worldwide.

The Republic of Singapore, situated in Southeast Asia, is an island city-state with a land area of 734.3 km^2^ and a population of 5.64 million [15,16]. Being highly urbanized, Singapore exhibits remarkable population density, with nearly 8000 people per km^2^ and is significantly denser in primary residential areas [17]. The majority of Singapore’s residents (78%) reside in high-rise public apartments. Serving as a prominent travel and business hub, Singapore attracted approximately 18.5 million visitors in 2018 [18], with the constant influx of travelers enabling the frequent emergence of new genotypes. Furthermore, the tropical climate is ideal for year-round breeding of the *Aedes aegypti* vector, facilitating endemic dengue transmission. However, due to a comprehensive vector control program, the force of infection has steadily decreased, concomitantly with dengue seroprevalence across all age groups [18,19]. This has led to a low level of herd immunity, particularly among the young [18], enabling explosive outbreaks to occur once dengue transmission takes hold.

Singapore first initiated a comprehensive nationwide program in 1969 to prevent and control the spread of DENV, which was fully implemented in 1973 [20]. The program encompassed various measures such as reducing mosquito breeding sites, conducting health education campaigns, and enforcing relevant laws and regulations. Presently, the primary approach is focused on preventative surveillance and larval source reduction, in both inter-epidemic and epidemic phases of dengue transmission, although new approaches to vector control are being tested [18,19]. Given that this approach is labor-intensive, relying on a limited number of skilled vector control officers, there is a pertinent need to understand which areas are at high-risk of dengue transmission to better allocate limited vector control resources.

Therefore, the primary objective of our study is to understand the environmental and anthropogenic factors which are associated with dengue transmission. First, we harmonized a high-dimensional and high-resolution repository of environmental and anthropogenic data together with comprehensively recorded dengue surveillance information over 2014–2020. We used generalized linear models and generalized additive models to delineate potential linear and non-linear associations between dengue transmission and potential risk factors. Furthermore, Shapley additive explanations were used to understand the contributions of each considered variable’s impact on dengue incidence rates. The risks of each environmental, anthropogenic, meteorological, and atmospheric factor on dengue incidence rates in each location were then converted into the incidence rate ratio scale for interpretation. Our study can inform urban planners, public health professionals, and policy makers on potential risk factors of dengue transmission.

## 2. Materials and Methods

### 2.1. Study Area and Dengue Data

Dengue is a legally notifiable disease in Singapore. All clinically diagnosed or laboratory confirmed cases of dengue in Singapore are legally required to be notified under the Infectious Disease Act [21]. In accordance with MOH criteria, individuals with confirmed DENV infection through RT-qPCR, positive NS1 antigen testing, or detection of IgM antibodies are classified as dengue cases. Following an epidemiological investigation to establish the location where the infection was contracted, the cases are linked to their corresponding spatial units. Spatial units typically comprise 10–20 public housing buildings each and are used for operational planning of vector control. Spatial units were stratified based on the primary type of housing found within each spatial unit, namely public and private housing. These were used as the primary units for two distinct analyses. Across the period of this study, a total of 8439 spatial units were used for our analyses, with 5611 spatial units belonging to the public housing category and 2828 spatial units belonging to the private housing category. Dengue case data was collected from Epidemiological Week (EW) 1 2007 to EW 52 2020. The annual case counts for each spatial unit were computed by aggregating weekly case counts. Annual dengue incidence rate, which serves as the primary outcome of this study, was obtained by dividing annual case counts by the population present in each spatial unit.

### 2.2. Exposures

A comprehensive set of spatio-temporal variables was collected to serve as indicators of environmental heterogeneity. A detailed description of the data sources and processing procedures for the variables can be found in the Supplementary Information. Exposure variables are described in Table 1.

### 2.3. Statistical Analysis

Generalized linear models (GLMs) were first used to model linear relationships between annual dengue incidence rates and the exposures considered in this study. Negative binomial models were used, as the outcome of interest was zero-inflated (Refer to Supplementary Information, Figure S1). In each instance, annual dengue incidence rates were regressed against mean annual values of the exposure present within each spatial unit to examine year-to-year associations between dengue incidence and exposures of interest. The assumption of linearity was then relaxed, and generalized additive models (GAMs) were utilized to detect potential non-linearities in associations between the exposures and outcome. GAMs were constructed using the *mgcv* package in R (Version 4.2.2). All variables included in the GAMs were smoothed using thin plate regression splines, as they offer a solution to the challenges associated with knot placement and have lower mean squared errors compared to knot-based splines in a pure regression context [22]. We utilized restricted maximum likelihood (REML) for smoothing parameter selection over alternatives such as generalized cross-validation (GCV), as such methods are prone to under-smoothing as well as over-fitting [23]. The log of the population was added as an offset term in both generalized linear models and generalized additive models to account for the differences in at-risk population within each spatial unit. As a sensitivity analysis, we employed backward stepwise regression to assess the goodness of fit of both GAMs and GLMs. Beginning with full models that included all exposures, we iteratively removed exposures in successive order, and each set of exposures was tested using the Akaike Information Criterion (AIC). This allowed us to observe which set of variables gives the most statistically significant improvement of the fit of each model to their corresponding datasets while penalizing model complexity [24] (Refer to Supplementary Information, Tables S7 and S8).

For GAMs, mean exposure-response curves were derived for a more intuitive interpretation of the impact of each covariate on dengue incidence rates. This method provides an estimate of the expected change in the incidence rates for a spatial unit given a particular value of the exposure of interest. We further computed an incidence rate ratio (IRR), which we defined as the ratio difference in dengue incidence rates given the values of an exposure of interest over the dengue incidence rates at the mean value of the exposure of interest. The IRR quantity was redefined as linearity assumptions in the GAM framework were relaxed. The IRR was computed by first predicting dengue incidence rates using the fitted GAM model at each value of the exposure of interest’s observed range while keeping all other exposures at their mean values. We then obtained IRR estimates by taking the numerator as the predicted dengue incidence rates at the varied values and the denominator at the predicted incidence rate value at the mean value of the exposure (Equation (1)),

(1) $$IRR=\frac{E\left[\left.\hat{Y}\right|\left(\;X_{i}=X_{ii}\;,\;\;X_{j}=\bar{X_{j}}\;\right)\;\right]}{E\left[\left.\hat{Y}\right|\left(\;X_{i}=\bar{X_{i}}\;,\;\;X_{j}=\bar{X_{j}}\;\right)\;\right]}$$

where $\hat{Y}$ is the estimated dengue incidence rates, $X_{i}$ is the exposure of interest, $X_{ii}$ is the ith value of the exposure of interest, and $X_{j}$ is the remaining set of exposures. Therefore, the IRR could be expressed as a factor by which the outcome—the dengue incidence rate—changes given the value of the exposure of interest compared to the incidence rate at the mean value of the same exposure of interest, while holding all other exposures at their mean values. As IRRs are a quantity that express a ratio difference, IRRs greater than one indicate a positive association, while IRRs less than one indicate a negative association. Confidence intervals (CIs) for IRR estimates were obtained by first generating 95% prediction intervals at each denominator and numerator value for the exposure of interest. The prediction intervals were then used to compute the upper and lower bounds of the IRR values for each denominator and numerator value.

To provide a separate interpretation of the importance of each exposure of interest on dengue incidence rates, we further computed Shapley additive explanations (SHAP). SHAP captures the impact of each exposure by quantifying how much it contributes to an individual prediction compared to the average prediction when combined with other exposures [25]. SHAP values were calculated for all features and instances across both datasets in this study. Although SHAP values are typically used to analyze local predictions, we employed two methods to combine local SHAP values into global explanations. Firstly, we computed the global importance of the features within each dataset. This was performed by averaging the absolute Shapley values per feature across the data. Mean absolute Shapley values indicate the absolute sum of all contributions towards predictions for an exposure across its entire range of observed values. This allows us to quantify the perceived importance of each exposure in our estimates of dengue incidence rates in comparison to the remaining exposures. Secondly, in order to observe both the magnitude and directions of the SHAP values simultaneously for each prediction instance, we plotted SHAP values against the observed range of values of each feature across all datasets.

### 2.4. Spatial Autocorrelation Analysis

We assessed the spatial autocorrelation in our outcome—annual dengue incidence rates—to examine the degree of similarity among values in neighboring spatial units. Specifically, we computed the Moran’s I statistic for our outcome and assessed its significance using a randomization procedure. This was achieved by repeatedly permuting the values of the variable being analyzed while preserving the spatial structure to generate a distribution of Moran’s I values under the assumption of spatial randomness. Furthermore, we also conducted Moran’s I analysis on our model residuals to ascertain that there was no retention of spatial autocorrelation structure in our models.

## 3. Results

### 3.1. Study Setting

Between the study period of EW 1 2014 and EW 52 2020, a total of 55,483 dengue case counts were reported from the spatial units in this study, with epidemiological year (EY) 2020 having the largest outbreak, culminating in 15,330 dengue cases (Refer to Supplementary Information, Figure S2). With the exception of NDVI, the vegetation related exposures exhibited a substantial spread across both data sets (Table 1). Mean values for the vegetation factors were similar across both datasets. Amongst anthropogenic exposures, the number of public housing units and number of condominium units displayed the greatest variation (Table 1). Meteorological exposures generally displayed little variation in values. The maximum values for total daily rainfall, mean temperature, and mean wind speed during the period of this study were 9.09 mm, 28.88 °C, and 13.36 km/h, respectively. Ambient air pollutant concentrations remained low throughout the study period as well, with little variation among observed values (Ranges: 21.04–39.38 mg/m^3^, 0.01–0.02 ppm, 6.55–27.56 ppb, 1.29–7.93 ppb, 0.23–0.44 ppm for PM_10_, O_3_, NO_2_, SO_2_, and CO, respectively).

### 3.2. Model Assesment

Comparison of GAM models with GLM alternatives demonstrate that GAMs provided the best fit to the data across all datasets (Refer to Supplementary Information, Tables S7 and S8). The differences in AIC between the model with all exposure variables included and the final model from the stepwise regression were found to be small. While the reduced-variable model from the stepwise regression offered a modest improvement in AIC, it risks the incorporation of biased coefficients. As the coefficients of the full models are unbiased, and considering the marginal improvement in AIC, it was not justifiable to opt for the reduced-variable models. Associations between dengue and exposure variables were interpreted using the IRR plots from the GAM models that included all variables as a result.

Moran’s I for the distribution of dengue incidence rates ranged from 0.10 to 0.36 for significant values in the public housing study setting and from 0.06 to 0.33 in the private housing study setting (Refer to Supplementary Information, Tables S1 and S2). However, Moran’s I test on the model residuals revealed Moran’s I test statistics between −0.02 and 0.03 for the public housing study setting and between −0.09 and 0.02 in the public housing study setting (Refer to Supplementary Information, Tables S3 and S4). All p-values were found to be non-significant, indicating the absence of spatial patterns in the model residuals.

### 3.3. Effect of Preceding Year Incidence Rate

**Figure 1:**
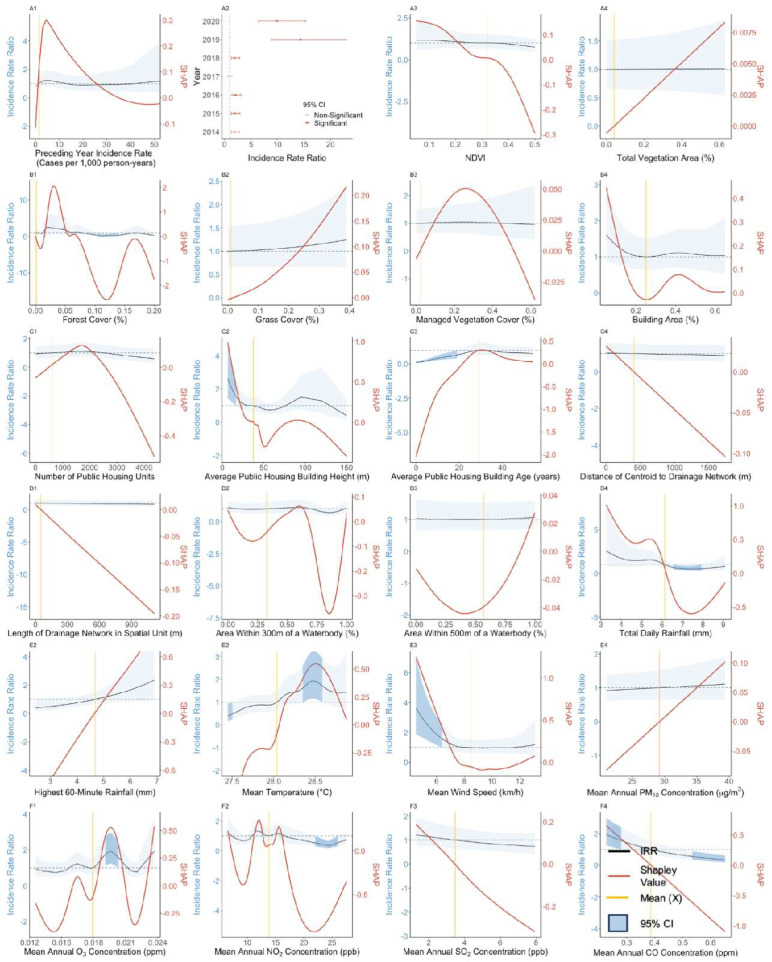
Exposure-response curves and SHAP values derived from public housing spatial units of preceding year incidence rate (**A1**), year (**A2**), normalized difference vegetation index (**A3**), total vegetation area (**A4**), forest cover (**B1**), grass cover (**B2**), managed vegetation cover (**B3**), building area (**B4**), number of public housing units (**C1**), average public housing building height (**C2**), average public housing building age (**C3**), distance of centroid to drainage (**C4**), length of drainage network in spatial unit (**D1**), area within 300 m of a water body (**D2**), area within 500 m of a water body (**D3**), total daily rainfall (**D4**), highest 60-min rainfall (**E1**), mean temperature (**E2**), mean wind speed (**E3**), mean annual PM_10_ concentration (**E4**), mean annual O_3_ concentration (**F1**), mean annual NO_2_ concentration (**F2**), mean annual SO_2_ concentration, (**F3**) and mean annual CO concentration (**F4**). Light blue shaded areas indicate the 95% confidence intervals. The red lines represent smoothed SHAP values, which indicate the predictive contribution of each exposure to dengue incidence rates. The black lines represent IRR estimates, indicating the factor change in dengue incidence rates across the observed range of the exposure of interest relative to the mean value of that exposure. The vertical golden line marks the mean value for the exposure of interest across its observed range in the dataset. The horizontal dashed grey line serves as a reference for an IRR estimate of one.

**Figure 2:**
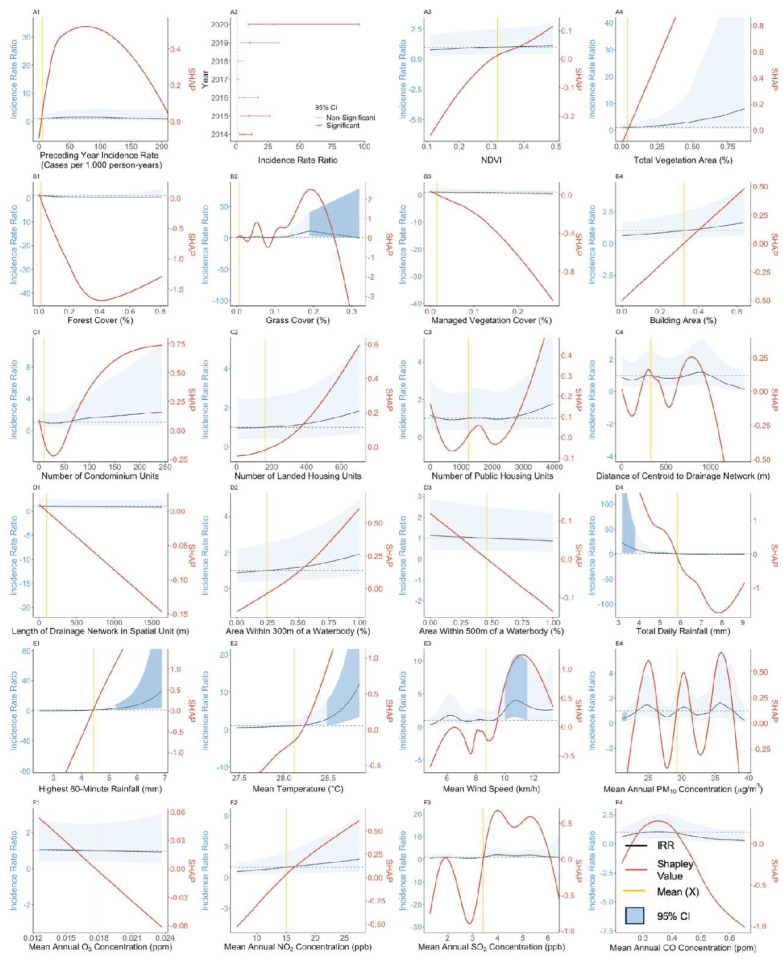
Exposure-response curves and SHAP values of preceding year case counts (**A1**), year (**A2**), normalized difference vegetation index (**A3**), total vegetation area (**A4**), forest cover (**B1**), grass cover (**B2**), managed vegetation cover (**B3**), building area (**B4**), number of condominium units (**C1**), number of landed housing units (**C2**), number of public housing units (**C3**), distance of centroid to drainage (**C4**), length of drainage network in spatial unit (**D1**), area within 300 m of a water body (**D2**), area within 500 m of a water body (**D3**), total daily rainfall (**D4**), highest 60-min rainfall (**E1**), mean temperature (**E2**), mean wind speed (**E3**), mean annual PM_10_ concentration (**E4**), mean annual O_3_ concentration (**F1**), mean annual NO_2_ concentration (**F2**), mean annual SO_2_ concentration, (**F3**) and mean annual CO concentration (**F4**). Light blue shaded areas indicate the 95% confidence intervals. The red lines represent smoothed SHAP values, which indicate the predictive contribution of each exposure to dengue incidence rates. The black lines represent IRR estimates, indicating the factor change in dengue incidence rates across the observed range of the exposure of interest relative to the mean value of that exposure. The vertical golden line marks the mean value for the exposure of interest across its observed range in the dataset. The horizontal dashed grey line serves as a reference for an IRR estimate of one.

**Figure 3:**
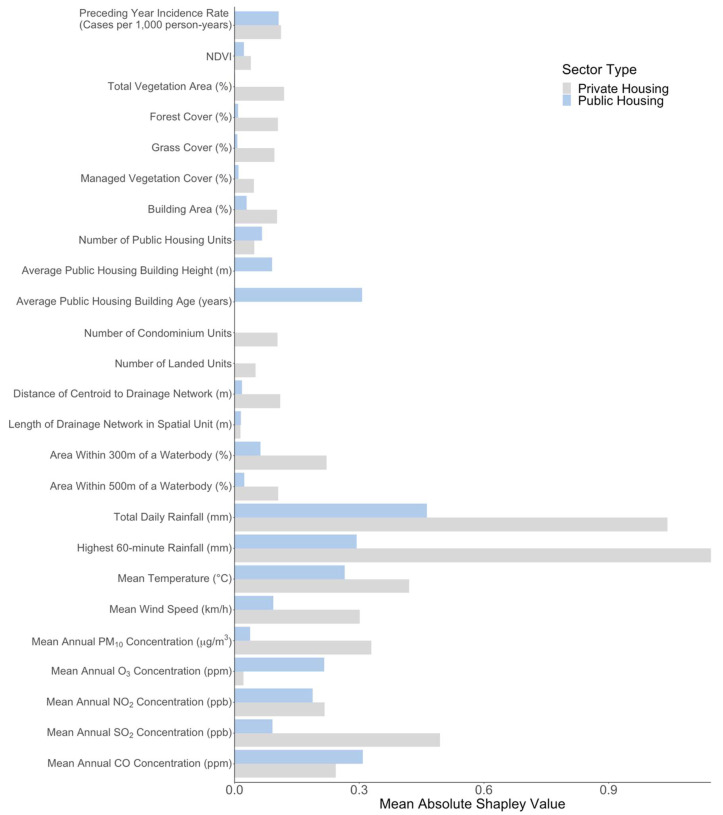
Mean absolute Shapley values of exposures across all spatial units. Light blue bars correspond to mean absolute Shapley values of the exposures in the public housing spatial units, while grey bars correspond to those in private housing spatial units.

Our results suggest a generally positive and non-linear exposure-response association between annual dengue incidence rates and preceding year incidence rates across both datasets (Figure 1(A1) and Figure 2(A1)). At preceding year incidence rate values larger than 0, IRR ranged from 0.80 (Figure 1(A1), 95% CI: 0.52–1.23) to 1.23 (Figure 1(A1), 95% CI: 0.79–1.92) for public housing spatial units. However, the association was found to be insignificant, as confidence intervals for the IRR overlap with one across all observed values in both types of spatial units. Mean absolute Shapley additive explanation (SHAP) values for preceding year dengue incidence rates were amongst the lowest for both private and public housing spatial units (Figure 3) in comparison to other exposures, indicating that this exposure afforded only a weak predictive contribution toward contemporaneous dengue incidence rates.

### 3.4. Effect of Vegetation-Related Exposures

NDVI, total vegetation area and managed vegetation cover, were estimated to have non-significant associations with dengue incidence rates. In the private housing study setting, forest cover above mean values were estimated to have a strong negative but non-significant association with dengue incidence rates, with IRR estimates ranging between 0.58 (Figure 2(B1), 95% CI: 0.20–1.52) and 0.19 (Figure 2(B1), 95% CI: 0.03–1.12) over the respective forest cover range of 11% to 42%. At this range, SHAP values indicated that forest cover had a strong negative predictive contribution toward the dengue incidence rate, ranging from −0.6 to −1.7 over the observed forest cover range. Grass cover exhibited a positive association with dengue incidence rates above mean values in the private housing spatial units, with an IRR estimate of 10.94 (Figure 2(B2), 95% CI: 2.91–41.04) at the observed grass cover value of 19%. However, both IRR estimates and SHAP values begin dropping sharply beyond this value, indicating that increasing grass cover beyond this point leads to a negative contribution toward predicted dengue incidence rates. Vegetation factors had mean absolute Shapley values below 0.15 (Figure 3), placing them towards the lower end of exposure importance, with total vegetation areas having the highest importance amongst the vegetation factors.

### 3.5. Effect of Anthropogenic Exposures

Building cover, number of public housing units, number of condominium units, number of landed units, and length of drainage network were found to have positive, albeit insignificant associations with dengue incidence across spatial units with either public or private housing. In general, only average public housing building height (Figure 1(C2)) and average public housing building age (Figure 1(C3)) were found to have significant associations with dengue incidence amongst the anthropogenic exposures. Average public housing building height exhibited a positive association with dengue incidence below mean values with heights between 6.5 m and 15 m, corresponding to IRR estimates between 2.62 (Figure 1(C2), 95% CI: 1.44–4.77) and 1.68 (Figure 1(C2), 95% CI: 1.05–2.73), respectively, compared to the reference mean public housing building height of 37.2 m. Conversely, average public building age below mean values was negatively associated with dengue incidence rates, compared to the reference mean public housing building age of 29.1 years. Building ages between 0.94 to 19.00 years corresponded with IRR estimates between 0.11 (Figure 1(C3), 95% CI: 0.07–0.19) and 0.65 (Figure 1(C3), 95% CI: 0.42–1.00). The mean absolute Shapley value for average public housing building age was 0.31, the highest amongst all anthropogenic exposures, indicating a strong contribution toward predicted dengue incidence rates. Average public building housing height had a mean absolute Shapley value of 0.09, indicating a weaker contribution toward dengue incidence rate predictions (Refer to Figure 3).

Mixed associations were observed between dengue incidence rates and areas within 300 m and 500 m of water bodies across both private housing and public housing spatial units. However, the associations were found to be insignificant across all observed range of values in this study. While SHAP values agreed with the trend of IRR estimates, the mean absolute Shapley values for both exposures were found to be below 0.25, indicating a weak contribution toward dengue incidence (Figure 3).

### 3.6. Effect of Meteorological and Atmospheric Exposures

Mean total daily rainfall was estimated to have a positive association with dengue incidence rates at values below the reference mean in the private housing study setting (Figure 2(D4)) and a negative association at values above the reference mean in the public housing study setting (Figure 1(D4)). IRR estimates below mean values of 5.85 mm were found to be significant, ranging from 22.75 (Figure 2(D4), 95% CI: 2.49–207.83) to 3.75 (Figure 2(D4), 95% CI: 1.00–14.05) as total daily rainfall increased from 3.16 mm to 4.40 mm. Mean temperature was significantly positively associated (Figure 1(E2) and Figure 2(E2)) with dengue incidence rates above the reference mean of 28.0 °C and 28.1 °C for the public and private housing spatial units, respectively, whereas mean temperature was negatively associated with dengue incidence rates when it was below mean reference values. For example, in the public housing study setting, IRR estimates for mean temperature ranged from 0.40 (Figure 1(E2), 95% CI: 0.17–0.94) to 0.51 (Figure 1(E2), 95% CI: 0.26–1.00) as temperatures increased from 27.4 °C to 27.5 °C, and ranged from 1.62 (Figure 1(E2), 95% CI: 1.00–2.62) to 1.72 (Figure 1(E2), 95% CI: 1.00–2.95) as temperatures increased from 28.3 °C to 28.6 °C.

Mean wind speed was estimated to have positive associations with dengue incidence below mean values in the public housing spatial units, with IRR estimates ranging from 3.65 (Figure 1(E3), 95% CI: 1.87–7.10) to 1.60 (Figure 1(E3), 95% CI: 1.00–2.55) as mean wind speed increased from 4.53 km/h to 6.40 km/h. The observed mean wind speed for the public housing dataset was 8.47 km/h. In cases where associations were significant, ambient air pollution exposures generally had positive associations with dengue incidence below mean values and a negative association above mean values. For example, in the public housing study setting, mean annual CO surface concentration was estimated to have an IRR ranging from 1.94 (Figure 1(F4), 95% CI: 1.25–3.01) to 1.44 (Figure 1(F4), 95% CI: 0.95–2.20) as its observed value ranged from 0.25 ppm to 0.30 ppm. Above its mean value of 0.39 ppm, the IRR was estimated to range from 0.65 (Figure 1(F4), 95% CI: 0.40–1.04) to 0.33 (Figure 1(F4), 95% CI: 0.18–0.61) as mean annual surface concentrations increased from 0.49 ppm to 0.65 ppm. Mean annual NO_2_ surface concentrations were estimated to have an IRR of 0.35 (Figure 1(F2), 95% CI: 0.18–0.66) at its observed value of 24.4 ppb in the public housing study setting, although its associations with dengue incidence were found to be insignificant at all other observed values.

Mean SHAP values were the greatest for total daily rainfall and highest 60-min rainfall across both study settings in comparison to all exposures considered in this study. Ambient air pollutant surface concentrations had generally consistent mean absolute SHAP values, falling between 0.18 to 0.49, with the exception of O_3_ and PM_10_, which had low values of 0.01 for the private housing spatial units (Figure 3) and 0.04 for public housing spatial units (Figure 3). Meteorological and atmospheric exposures typically exhibited greater contributions towards dengue incidence rate estimates in comparison to vegetation and anthropogenic exposures (Figure 3).

## 4. Discussion

Our study examined the associations between dengue incidence rates and a wide range of anthropogenic and ecological exposures. These associations were studied over a fine spatial scale, consisting of 1259 spatial units within Singapore over a period of 7 years. The associations were studied by sub-setting our datasets into the primary type of housing found in each spatial unit, with associations for each exposure estimated to be generally consistent across datasets. The findings obtained from this study build upon previous work [26,27,28,29] that sought to establish a thorough understanding of DENV transmission.

Our study found a negative association between vegetation factors and dengue incidence rates in general (Figure 1(A3–B3) and Figure 2(A3–B3)). Vegetation factors included NDVI, which is a measure of vegetation that combines the impact of vegetation quantity, including its coverage and biomass, and quality [30], as well as total vegetation area, forest cover, managed vegetation cover, and grass cover. The negative association between vegetation cover and dengue transmission is consistent with previous studies [31,32] that have shown that variables such as farm, forest, and grassland have significant negative correlations with dengue transmission and can provide a protective barrier against *Ae. aegypti* populations. Locally, an analysis of dengue vector populations found that forest cover was associated with a 7.4% decrease in *Aedes* abundance per standard deviation increase of forest cover [26], providing a possible explanation for the reduction in dengue incidence rates observed when forest cover values were above the mean. The findings are unsurprising, as *Ae. aegypti* has been documented to exhibit a strong preference for urban environments [8], residing within human dwellings or in close association with human habitation, and laying eggs in human-made containers. While the associations between vegetation and dengue incidence were found to be significant in the aforementioned studies, our study only found a significant relationship between grass cover and dengue incidence. Transient puddles, typically encircled by short grasses, have been identified as optimal natural breeding sites for *Anopheles gambiae*/*Anopheles coluzzii* and *Anopheles arabiensis* [33]. These puddles could similarly provide breeding sites for *Aedes* larvae, which could serve as a potential explanation for increased dengue incidence rates in regions with greater grass cover. The difference in the significance of vegetation-related factors between our study and others could be due to several reasons, such as the differing spatial resolutions of our analysis, non-standard categorization of vegetation covers, or the inherent complexity of the associations between dengue incidence and vegetation-related exposures.

Average public housing building height was estimated to have a significant positive association below mean values in this study (Figure 1(C2)), which corroborates with the findings of other studies that explored the effects of the patterns of urban housing on dengue distribution [34]. In particular, urban drainage structures, such as storm drains and gully traps, which retain rainwater and runoff, offer a conducive breeding ground for immature *Aedes* spp. [35,36,37], potentially driving up vector populations, and consequentially, dengue incidence rates. Another viable explanation is that the dispersal of mosquito species such as *Aedes* that feed on mammals tends to be low [38]. Factors such as the physiological status of the female, body size, and flight strength influence their flight patterns, resulting in females flying at lower heights, just above the top of vegetation. The exposure of hosts to the vector is therefore likely to be more prevalent for those living in low-rise buildings, resulting in the higher predicted dengue incidence rates.

Another anthropogenic exposure that was estimated to have a significant association with dengue incidence was average public housing building age. Public housing buildings with ages below mean values were estimated to have less than half the dengue incidence rates compared to those above mean values (Figure 1(C3), IRR: 0.30, 95% CI: 0.19–0.47, Average Public Housing Building Height: 7.25 m). This could be due to multiple factors, such as poorer infrastructure design in older buildings and deterioration of public housing infrastructure over time. Another study has shown that older building age was associated with increased *Aedes* abundance in Singapore [26], with a 52.3% increase in *Aedes aegypti* abundance per 10-year increase in average building age, thus contributing to an increased risk of dengue transmission.

Multiple studies within Southeast Asia have shown that temperature is a significant climate variable [39,40] associated with DENV transmission. We estimated that mean temperatures above 28 °C were positively associated with dengue incidence rates (Figure 1(E2)), while mean temperatures below 28 °C were negatively associated with dengue incidence rates. These findings align with the concept of an optimal temperature range that facilitates dengue transmission, while temperatures beyond this range may hinder the transmission of dengue virus. Previous studies have found varying associations between temperature and dengue incidence in Singapore. A longitudinal study between 1974 and 2011 found that higher mean temperatures were correlated with higher DENV transmission [41]. Xu et al. (2014) found that dengue cases in Singapore were more prevalent when the mean temperatures exceeded the reference value of 27.8 °C during the period of 2001–2009. However, all values outside of the reference mean temperature of 27.8 °C in the years 2004–2006 and 2007–2009 were linked to a decrease in dengue transmission [42]. Possible explanations for these discrepancies could be the differing effects of temperature across the mosquito’s natural developmental cycles and the need to account for a lag period for these effects to manifest, which is difficult to estimate when considering the resolution of our analysis, which is on an annual time-frame.

The significant positive association with wind speed below mean values predicted in this study (Figure 1(E3)) aligns with a study conducted in Guangzhou, China, where an inverse relationship between wind velocity and dengue incidence was reported within the same month [43]. This could potentially be attributed to the inhibition of mosquitoes’ flight activity in search of hosts [44], resulting in a decrease in oviposition and reduced contact between the vector and hosts. The presence of wind can also hinder mosquitoes from tracking scent plumes [45] due to their inability to progress upwind, or because the chemical attractants released by the host become diluted and dispersed. Moreover, wind also has a direct impact on the rate of evaporation for both outdoor and indoor breeding sites of vectors [46], which may reduce aquatic carrying capacity.

The relationship between rainfall and dengue incidence has exhibited a wide range of associations, varying from weak or negligible [47,48] to as much as a 21% increase in dengue incidence in response to heightened rainfall [44]. We estimated positive associations between dengue incidence and mean total daily rainfall below mean values among private housing spatial units (Figure 2(D4)), while associations between dengue incidence and mean total daily rainfall in the public housing spatial units were estimated to be positive above the mean value of 6.18 mm (Figure 1(D4)). Our study agrees with the findings of other studies. While rain is a recognized risk factor for dengue incidence [49], a longitudinal study examining dengue cases in Singapore [50] spanning from 2000 to 2007 revealed no correlation between rainfall and dengue incidence. One possible explanation for this could be the abundance of indoor breeding sights in large urban centers, offering a sheltered environment that remains unaffected by outdoor elements. A study in Singapore [51] also demonstrated that excessive rainfall has the potential to eliminate breeding sites and disrupt the development of larvae through a process described as ‘flushing out’, which may explain the negative association between total daily rainfall and dengue incidence rates in our study.

Ambient air pollutant surface concentrations above mean values were predicted to have a negative association with dengue incidence rates in our analysis. This effect was found to be especially profound regarding mean annual NO_2_ concentrations (Figure 1(F2)), with values above the mean resulting in a 65% decrease in dengue incidence rates (IRR: 0.35, 95% CI: 0.18–0.66, Mean Annual NO_2_ Concentration: 24.4 ppb). These findings are consistent with other studies that have investigated the effects of pollutants on vectors’ biological mechanisms. Insects are known to experience harmful or potentially fatal effects from pollutants, pesticides, heavy metals, and fine particulate matter, which originate from industrial activities found in urban centers [52]. Such pollutants have been shown to strain the vector’s biological mechanisms, affecting their behavior such as feeding and breeding cycles. Phanichat et al. (2021) demonstrated that *Ae. aegypti* exhibit decreased blood-feeding activity in response to elevated levels of PM_2.5_ exposure [53], possibly due to pollutant’s effect on their olfactory system. These effects may not be limited to particulate matter and could potentially be extended to air pollutants in general.

When interpreting our findings, it is important to consider the following limitations. Firstly, we were unable to incorporate the impact of vector control programs that are conducted within the spatial units. Activities such as regulatory inspections and community interventions to eliminate mosquito breeding sites, along with chemical control methods used to reduce adult mosquito populations tend to be more intensive within regions where higher case counts are detected. This could obscure the true relationship between our exposures of interest, and the extent of this confounding bias is challenging to account for [54]. Spatial units where *Wolbachia* intervention was carried out as a part of the NEA’s vector control program were excluded from this study, as the program has been found to drastically reduce dengue incidence in Singapore [54]. Secondly, the effect of meteorological exposures, which were shown in our study to be the strongest predictors, typically manifest within weeks, and our predicted estimates on an annual time-scale would be unable to accurately capture these trends. Thirdly, as spatial units may contain both types of housing, there is a possibility of a case where an infection is contracted by someone who resides in public housing yet belongs to a spatial unit that is primarily private housing, and vice versa, which could lead to us wrongly attributing associations to the wrong variables. However, as our spatial resolution is high, such a case is unlikely. The primary limitation of this study is its lack of generalizability over different geographical regions. While Singapore benefits from a comprehensive case notification system that facilitates this data availability, it may pose challenges in jurisdictions that lack the small size and well-defined boundaries of Singapore’s population. Finally, while a robust sensitivity analysis and literature review was conducted before selecting our exposures, our study is still prone to omitted variable bias. This could potentially lead to our model incorrectly attributing the effects of the omitted variables to the exposures chosen in this study.

In conclusion, the analyses presented in this study provide valuable insights into the complex dynamics of dengue incidence in Singapore. By combining these predictive insights with effective control measures, public health authorities can proactively identify high-risk areas and allocate resources strategically for optimal vector control efforts. The insights can also be used for effective land use planning that maximizes the protective effect of the urban environment against dengue transmission. As dengue dynamics evolve, refinement of our models can be achieved through the inclusion of relevant variables such as population mobility, comprehensive data on vector control programs, and local environment changes, contributing to their robustness. Our study underscores the importance of an interdisciplinary approach to combat dengue incidence, leveraging upon the power of predictive modelling, informed decision making and evidence-based control measures.

## Figures and Tables

**Table 1:** Summary statistics of variables included in the study.

			Mean (*SD*)	
	Exposure	Description	Public Housing Spatial Units	Private Housing Spatial Units
	Current Year Incidence Rate (Cases per 1000 person-years)		1.57 (4.54)	7.43 (19.57)
	Population		5103.62 (2661.30)	949.00 (852.25)
	Preceding Year Incidence Rate (Cases per 1000 person-years)		1.45 (4.41)	5.86 (18.11)
Vegetation-Related	NDVI	NDVI serves as an alternative measure of vegetation cover.	0.32 (0.06)	0.32 (0.06)
	Total Vegetation Area (%)	Percentage cover of vegetation exposures, with areas classified across multiple vegetation types including grass, forest, and managed vegetation. Managed Vegetation was defined as vegetation found in park areas.	0.04 (0.08)	0.04 (0.09)
	Forest Cover (%)		0.001 (0.01)	0.01 (0.07)
	Grass Cover (%)		0.01 (0.04)	0.01 (0.03)
	Managed Vegetation Cover (%)		0.02 (0.05)	0.02 (0.03)
Anthropogenic	Building Area (%)	The percentage cover of built area was calculated by summing the areas of all commercial, industrial, and residential buildings, and serves as a measure of urbanicity.	0.25 (0.07)	0.32 (0.09)
	Number of Public Housing Units	Data on locations of public housing estates, representing the residences of most of the population.	609.77 (719.96)	1229.55 (677.14)
	Average Public Housing Building Height (m)	Average public housing building height was determined based on the number of floors and an assumed average height of 3 m per floor.	37.56 (12.32)	
	Average Public Housing Building Age (years)	Average age of buildings within each spatial unit was collected through residential location and resale data.	29.10 (11.98)	
	Number of Condominium Units	The number of condominiums and landed properties was collected for each spatial unit.		10.00 (19.43)
	Number of Landed Housing Units			161.67 (122.21)
	Distance of Centroid to Drainage Network (m)	Data on the major open drainage network in Singapore were collected from the Public Utilities Board, and were used to measure the distance between each HDB block and a drain, as well as the length of the drainage network present within the spatial unit.	407.20 (289.54)	325.61 (226.01)
	Length of Drainage Network in Spatial Unit (m)		50.47 (130.61)	103.26 (237.07)
	Area Within 300 m of a Waterbody (%)	Area within spatial units that fall within 300 m and 500 m radius buffers around waterbodies.	0.33 (0.40)	0.25 (0.36)
	Area Within 500 m of a Waterbody (%)		0.57 (0.44)	0.46 (0.44)
Meteorological	Total Daily Rainfall (mm)	Meteorological exposures that have been well established to influence DENV transmission were obtained from 21 local weather stations. As these variables were collected on a daily timescale, the values were harmonized to the annual timescale of the dengue incidence rates by taking the annual mean of respective exposure within each spatial unit.	6.13 (1.16)	5.85 (1.27)
	Highest 60-Minute Rainfall (mm)		4.68 (0.88)	4.44 (0.95)
	Mean Temperature (°C)		28.04 (0.26)	28.11 (0.26)
	Mean Wind Speed (km/h)		8.47 (1.20)	8.68 (1.10)
Atmospheric	Mean Annual PM_10_ Concentration (µg/m^3^)	Surface concentrations of ambient air pollutants were obtained from the Air Quality Open Data Platform, which compiles values provided by Singapore’s National Environment Agency (NEA). These values were also aggregated at an annual level to correspond with DENV data.	29.27 (4.46)	29.30 (4.50)
	Mean Annual O_3_ Concentration (ppm)		0.02 (0.002)	0.02 (0.002)
	Mean Annual NO_2_ Concentration (ppb)		13.81 (4.60)	15.02 (5.33)
	Mean Annual SO_2_ Concentration (ppb)		3.48 (1.34)	3.41 (1.22)
	Mean Annual CO Concentration (ppm)		0.39 (0.09)	0.39 (0.10)
Observations			5611	2828

## Data Availability

All code required to reproduce this study is available at https://github.com/prnvtwr/Dengue, accessed on 28 August 2023.

## Supplementary Materials

The following supporting information can be downloaded at: https://www.mdpi.com/article/10.3390/v15091917/s1, Figure S1: Distribution of Dengue Incidence Rates; Figure S2: Total Number of Reported Dengue Cases by Year; Table S1: Moran’s I Test Results of Dengue Incidence Rates of Public Housing Study Setting (2014–2020); Table S2: Moran’s I Test Results of Dengue Incidence Rates of Private Housing Study Setting (2014–2020); Table S3: Moran’s I Test Results of Model Residuals of Public Housing Study Setting (2014–2020); Table S4: Moran’s I Test Results of Model Residuals of Private Housing Study Setting (2014–2020); Table S5: Summary Statistics of Public Housing Study Setting (2014–2020); Table S6: Summary Statistics of Private Housing Study Setting (2014–2020); Table S7: Sensitivity Analysis of Public Housing Study Setting (2014–2020); Table S8: Sensitivity Analysis of Private Housing Study Setting (2014–2020); Table S9: Regression Results of Public Housing Study Setting (2014–2020); Table S10: Regression Results of Private Housing Study Setting (2014–2020); Table S11: Summary Statistics of Public Housing Study Setting (2008–2020); Table S12: Summary Statistics of Private Housing Study Setting (2008–2020); Table S13: Sensitivity Analysis of Public Housing Study Setting (2008–2020); Table S14: Sensitivity Analysis of Private Housing Study Setting (2008–2020); Table S15: Regression Results of Public Housing Study Setting (2008–2020); Table S16: Regression Results of Private Housing Study Setting (2008–2020). References [55,56,57,58,59,60] are listed in the supplementary materials.

## References

[1] Bhatt S., Gething P.W., Brady O.J., Messina J.P., Farlow A.W., Moyes C.L., Drake J.M., Brownstein J.S., Hoen A.G., Sankoh O. (2013). The global distribution and burden of dengue. Nature.

[2] Zeng Z., Zhan J., Chen L., Chen H., Cheng S. (2021). Global, regional, and national dengue burden from 1990 to 2017: A systematic analysis based on the global burden of disease study 2017. EClinicalMedicine.

[3] Messina J.P., Brady O.J., Golding N., Kraemer M.U.G., Wint G.R.W., Ray S.E., Pigott D.M., Shearer F.M., Johnson K., Earl L. (2019). The current and future global distribution and population at risk of dengue. Nat. Microbiol..

[4] Kolimenakis A., Heinz S., Wilson M.L., Winkler V., Yakob L., Michaelakis A., Papachristos D., Richardson C., Horstick O. (2021). The role of urbanisation in the spread of Aedes mosquitoes and the diseases they transmit—A systematic review. PLoS Negl. Trop. Dis..

[5] Kulkarni M.A., Duguay C., Ost K. (2022). Charting the evidence for climate change impacts on the global spread of malaria and dengue and adaptive responses: A scoping review of reviews. Glob. Health.

[6] Watts M.J., Kotsila P., Mortyn P.G., Sarto i Monteys V., Brancati C.U. (2020). Influence of socio-economic, demographic and climate factors on the regional distribution of dengue in the United States and Mexico. Int. J. Health Geogr..

[7] Buhler C., Winkler V., Runge-Ranzinger S., Boyce R., Horstick O. (2019). Environmental methods for dengue vector control—A systematic review and meta-analysis. PLoS Negl. Trop. Dis..

[8] Gubler D.J. (2011). Dengue, Urbanization and Globalization: The Unholy Trinity of the 21st Century. Trop. Med. Health.

[9] Harapan H., Michie A., Sasmono R.T., Imrie A. (2020). Dengue: A Minireview. Viruses.

[10] World Health Organization (2012). Global Strategy for Dengue Prevention and Control 2012–2020.

[11] Redoni M., Yacoub S., Rivino L., Giacobbe D.R., Luzzati R., Di Bella S. (2020). Dengue: Status of current and under-development vaccines. Rev. Med. Virol..

[12] Torres-Flores J.M., Reyes-Sandoval A., Salazar M.I. (2022). Dengue Vaccines: An Update. BioDrugs.

[13] EMA (2022). Qdenga. European Medicines Agency. https://www.ema.europa.eu/en/medicines/human/EPAR/qdenga.

[14] Wilson A.L., Courtenay O., Kelly-Hope L.A., Scott T.W., Takken W., Torr S.J., Lindsay S.W. (2020). The importance of vector control for the control and elimination of vector-borne diseases. PLoS Negl. Trop. Dis..

[15] Population Trends. http://www.singstat.gov.sg/publications/population/population-trends.

[16] Environment. http://www.singstat.gov.sg/find-data/search-by-theme/society/environment/latest-data.

[17] The World Bank World Bank Open Data. https://data.worldbank.org.

[18] Sim S., Ng L.C., Lindsay S.W., Wilson A.L. (2020). A greener vision for vector control: The example of the Singapore dengue control programme. PLoS Negl. Trop. Dis..

[19] Ho S.H., Lim J.T., Ong J., Hapuarachchi H.C., Sim S., Ng L.C. (2023). Singapore’s 5 decades of dengue prevention and control—Implications for global dengue control. PLoS Negl. Trop. Dis..

[20] Koh B.K., Ng L.C., Kita Y., Tang C.S., Ang L.W., Wong K.Y., James L., Goh K.T. (2008). The 2005 dengue epidemic in Singapore: Epidemiology, prevention and control. Ann. Acad. Med. Singap..

[21] MOH|Infectious Diseases Act. https://www.moh.gov.sg/policies-and-legislation/infectious-diseases-act.

[22] Wood S.N. (2003). Thin plate regression splines. J. R. Stat. Soc. Ser. B Stat. Methodol..

[23] Wood S.N. (2010). Fast Stable Restricted Maximum Likelihood and Marginal Likelihood Estimation of Semiparametric Generalized Linear Models. J. R. Stat. Soc. Ser. B Stat. Methodol..

[24] Akaike H., Petrov B.N., Csáki F. (1973). Information Theory and an Extension of the Maximum Likelihood Principle. 2nd International Symposium on Information Theory.

[25] Lundberg S., Lee S.I. (2017). A Unified Approach to Interpreting Model Predictions. arXiv.

[26] Sun H., Dickens B.L., Richards D., Ong J., Rajarethinam J., Hassim M.E.E., Lim J.T., Carrasco L.R., Aik J., Yap G. (2021). Spatio-temporal analysis of the main dengue vector populations in Singapore. Parasites Vectors.

[27] Soh S., Ho S.H., Seah A., Ong J., Richards D.R., Gaw L.Y.-F., Dickens B.S., Tan K.W., Koo J.R., Cook A.R. (2022). Spatial Methods for Inferring Extremes in Dengue Outbreak Risk in Singapore. Viruses.

[28] Ong J., Soh S., Ho S.H., Seah A., Dickens B.S., Tan K.W., Koo J.R., Cook A.R., Richards D.R., Gaw L.Y.-F. (2022). Fine-scale estimation of effective reproduction numbers for dengue surveillance. PLoS Comput. Biol..

[29] Lim J.T., Han Y., Dickens B.S.L., Choo E.L.W., Chew L.Z.X., Cook A.R. (2020). Revealing two dynamic dengue epidemic clusters in Thailand. BMC Infect. Dis..

[30] da Consolação Magalhães Cunha M., Ju Y., Morais M.H.F., Dronova I., Ribeiro S.P., Bruhn F.R.P., Lima L.L., Sales D.M., Schultes O.L., Rodriguez D.A. (2021). Disentangling associations between vegetation greenness and dengue in a Latin American city: Findings and challenges. Landsc. Urban Plan..

[31] Sari S.Y.I., Adelwin Y., Rinawan F.R. (2020). Land Use Changes and Cluster Identification of Dengue Hemorrhagic Fever Cases in Bandung, Indonesia. Trop. Med. Infect. Dis..

[32] Huang C.-C., Tam T.Y.T., Chern Y.-R., Lung S.-C.C., Chen N.-T., Wu C.-D. (2018). Spatial Clustering of Dengue Fever Incidence and Its Association with Surrounding Greenness. Int. J. Environ. Res. Public Health.

[33] Asmare Y., Hill S.R., Hopkins R.J., Tekie H., Ignell R. (2017). The role of grass volatiles on oviposition site selection by *Anopheles arabiensis* and *Anopheles coluzzii*. Malar. J..

[34] Seidahmed O.M.E., Lu D., Chong C.S., Ng L.C., Eltahir E.A.B. (2018). Patterns of Urban Housing Shape Dengue Distribution in Singapore at Neighborhood and Country Scales. GeoHealth.

[35] Montgomery B.L., Ritchie S.A., Hart A.J., Long S.A., Walsh I.D. (2004). Subsoil drain sumps are a key container for *Aedes aegypti* in Cairns, Australia. J. Am. Mosq. Control Assoc..

[36] Paploski I.A.D., Rodrigues M.S., Mugabe V.A., Kikuti M., Tavares A.S., Reis M.G., Kitron U., Ribeiro G.S. (2016). Storm drains as larval development and adult resting sites for *Aedes aegypti* and *Aedes albopictus* in Salvador, Brazil. Parasites Vectors.

[37] Fernandez S.A., Sun H., Dickens B.L., Ng L.C., Cook A.R., Lim J.T. (2023). Features of the urban environment associated with Aedes aegypti abundance in high-rise public apartments in Singapore: An environmental case-control study. PLoS Negl. Trop. Dis..

[38] Verdonschot P.F.M., Besse-Lototskaya A.A. (2014). Flight distance of mosquitoes (Culicidae): A metadata analysis to support the management of barrier zones around rewetted and newly constructed wetlands. Limnologica.

[39] Francisco M.E., Carvajal T.M., Ryo M., Nukazawa K., Amalin D.M., Watanabe K. (2021). Dengue disease dynamics are modulated by the combined influences of precipitation and landscape: A machine learning approach. Sci. Total Environ..

[40] Polwiang S. (2020). The time series seasonal patterns of dengue fever and associated weather variables in Bangkok (2003–2017). BMC Infect. Dis..

[41] Struchiner C.J., Rocklöv J., Wilder-Smith A., Massad E. (2015). Increasing Dengue Incidence in Singapore over the Past 40 Years: Population Growth, Climate and Mobility. PLoS ONE.

[42] Xu H.-Y., Fu X., Lee L.K.H., Ma S., Goh K.T., Wong J., Habibullah M.S., Lee G.K.K., Lim T.K., Tambyah P.A. (2014). Statistical Modeling Reveals the Effect of Absolute Humidity on Dengue in Singapore. PLoS Negl. Trop. Dis..

[43] Lu L., Lin H., Tian L., Yang W., Sun J., Liu Q. (2009). Time series analysis of dengue fever and weather in Guangzhou, China. BMC Public Health.

[44] Cheong Y.L., Burkart K., Leitão P.J., Lakes T. (2013). Assessing Weather Effects on Dengue Disease in Malaysia. Int. J. Environ. Res. Public Health.

[45] Hoffmann E.J., Miller J.R. (2002). Reduction of mosquito (Diptera: Culicidae) attacks on a human subject by combination of wind and vapor-phase DEET repellent. J. Med. Èntomol..

[46] Ehelepola N.D.B., Ariyaratne K., Buddhadasa W.M.N.P., Ratnayake S., Wickramasinghe M. (2015). A study of the correlation between dengue and weather in Kandy City, Sri Lanka (2003–2012) and lessons learned. Infect. Dis. Poverty.

[47] Goto K., Kumarendran B., Mettananda S., Gunasekara D., Fujii Y., Kaneko S. (2013). Analysis of Effects of Meteorological Factors on Dengue Incidence in Sri Lanka Using Time Series Data. PLoS ONE.

[48] Fan J., Lin H., Wang C., Bai L., Yang S., Chu C., Yang W., Liu Q. (2013). Identifying the high-risk areas and associated meteorological factors of dengue transmission in Guangdong Province, China from 2005 to 2011. Epidemiol. Infect..

[49] Li Y., Dou Q., Lu Y., Xiang H., Yu X., Liu S. (2020). Effects of ambient temperature and precipitation on the risk of dengue fever: A systematic review and updated meta-analysis. Environ. Res..

[50] Pinto E., Coelho M., Oliver L., Massad E. (2011). The influence of climate variables on dengue in Singapore. Int. J. Environ. Health Res..

[51] Benedum C.M., Seidahmed O.M.E., Eltahir E.A.B., Markuzon N. (2018). Statistical modeling of the effect of rainfall flushing on dengue transmission in Singapore. PLoS Negl. Trop. Dis..

[52] Feldhaar H., Otti O. (2020). Pollutants and Their Interaction with Diseases of Social Hymenoptera. Insects.

[53] Phanitchat T., Ampawong S., Yawootti A., Denpetkul T., Wadmanee N., Sompornrattanaphan M., Sivakorn C. (2021). Dose-Dependent Blood-Feeding Activity and Ovarian Alterations to PM2.5 in Aedes aegypti. Insects.

[54] Soh S., Ho S.H., Seah A., Ong J., Dickens B.S., Tan K.W., Koo J.R., Cook A.R., Tan K.B., Sim S. (2021). Economic impact of dengue in Singapore from 2010 to 2020 and the cost-effectiveness of Wolbachia interventions. PLoS Glob. Public Health.

[55] Gaw L.Y.-F., Yee A.T.K., Richards D.R. (2019). A high-resolution map of singapore’s terrestrial ecosystems. Data.

[56] (2023). USGS. https://www.usgs.gov/landsat-missions/landsat-normalized-difference-vegetation-index.

[57] OneMap (2023). Onemap Api. https://www.onemap.gov.sg/main/v2/.

[58] Technical Assistance Document for the Reporting of Daily Air Quality—The Air Quality Index (AQI). https://www.airnow.gov/sites/default/files/2020-05/aqi-technical-assistance-document-sept2018.pdf.

[59] ECMWF (2023). Resale Flat Prices. https://www.ecmwf.int/.

[60] DoS (2023). Singapore Department of Statistics (dos). https://www.singstat.gov.sg/.

