# Supplementary material for: Associations between Dengue Incidence, Ecological Factors, and Anthropogenic Factors in Singapore
Source: Viruses. 2023 Sep 13;15(9):1917. doi: 10.3390/v15091917 (PMC10535411; doi:10.3390/v15091917)
Supplement: Supplementary file 1 [file viruses-15-01917-s001.zip › viruses-2540020-supplementary.pdf]

Supplementary Information for *“Associations between dengue incidence, ecological and anthropogenic factors transmission in Singapore”*

Table of Contents

1 Supplementary Figures 2

1.1 Distribution of Incidence Rates 2

1.2 Total Number of Reported Cases by Year 3

2 Data 4

2.1 Vegetation Data 4

2.2 Residential Data 4

2.3 Meteorological Data 4

2.4 Air Pollutant Data 5

3. Analysis of Dengue Incidence Rates between 2014 – 2020 6

3.1 Spatial Autocorrelation 6

3.2 Summary Statistics 8

3.2 Sensitivity Analysis 10

3.3 Regression Outputs from Linear Models 14

4. Analysis of Dengue Incidence Rates from 2008 to 2020 19

4.1 Study Setting 19

4.2 Summary Statistics 19

4.3 Sensitivity Analysis 21

4.4 Regression Outputs from Linear Models 24

References 27

1 Supplementary Figures

1.1 Distribution of Incidence Rates

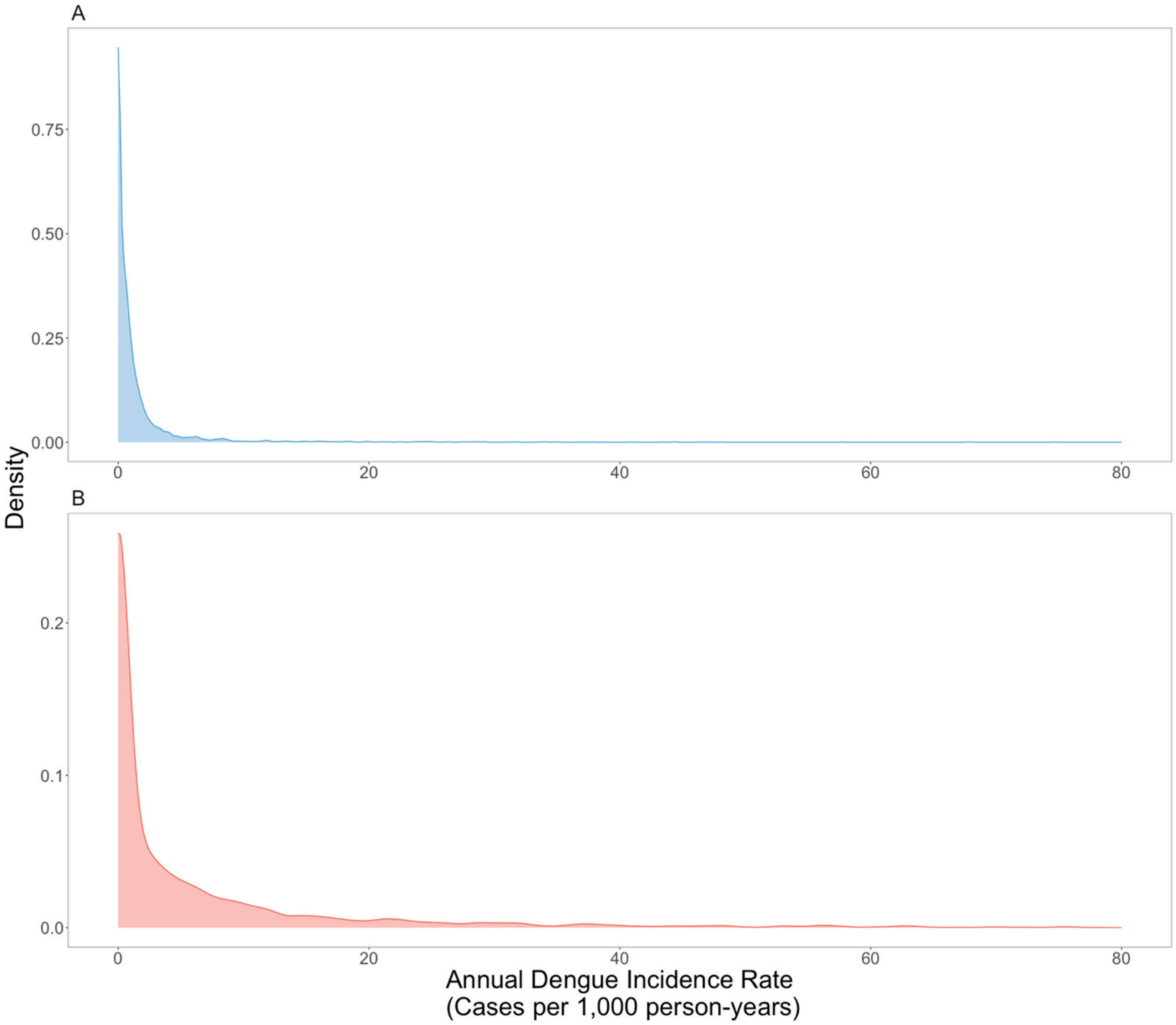

Figure S1 *Kernel density estimates of Annual Dengue Incidence Rates (Cases per 1,000 person-years) in (A) public housing spatial units (n = 5611) and (B) private housing spatial units (n = 2828). Concentrated densities near zero indicate that incidence rates are zero-inflated.*

1.2 Total Number of Reported Cases by Year

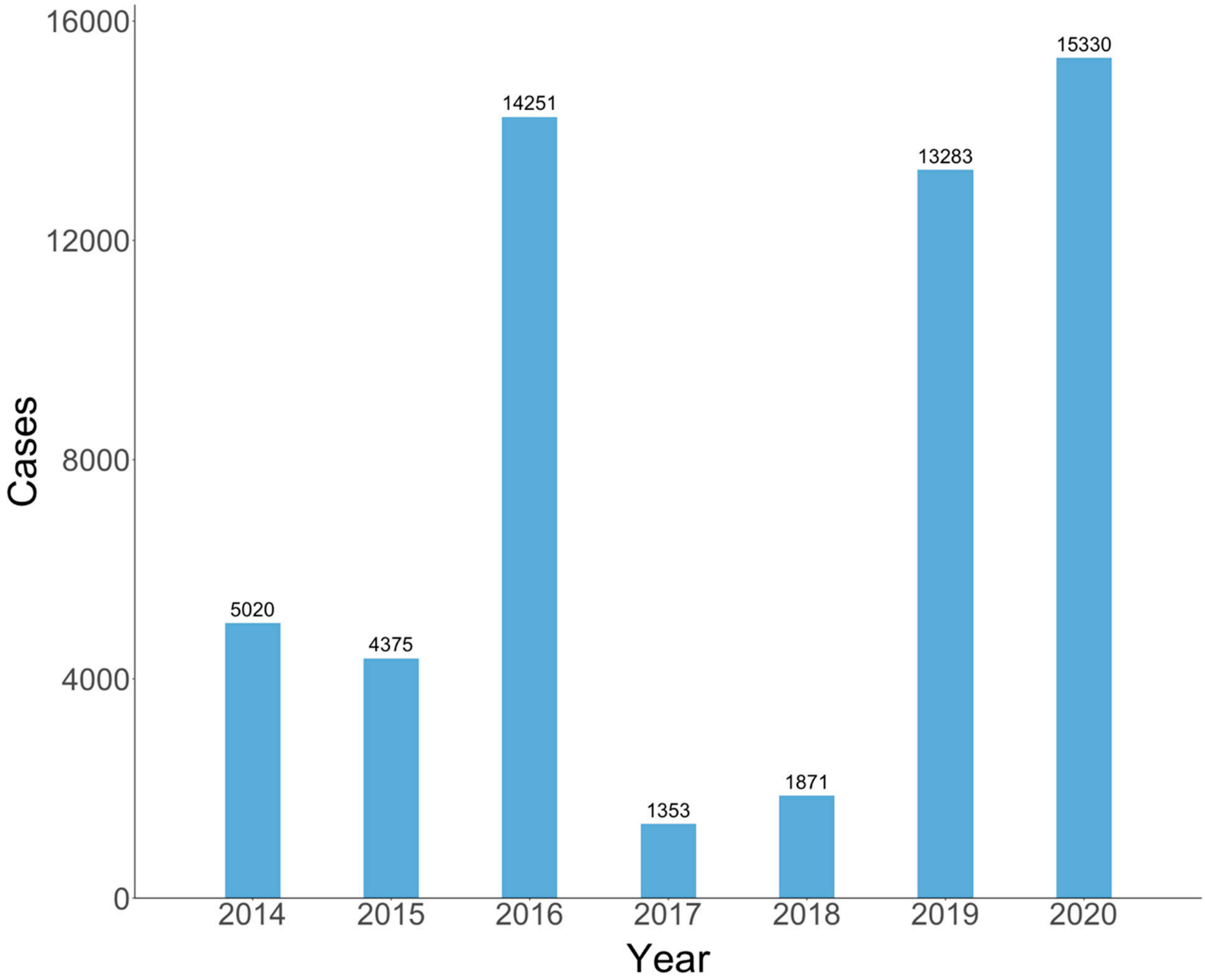

Figure S2 *Total number of reported dengue cases across all spatial units (n = 8,439) between the study period of 2014 and 2020. A total of 55,483 cases were reported during this period, with the highest number of annual cases being recorded in 2020.*

2 Data

We extracted a set of spatially explicit variables to represent environmental heterogeneity across sectors. Summary statistics for spatio-temporally resolved variables are provided in Tables S1 and S2.

2.1 Vegetation Data

A 10m vegetation map (Gaw et al., 2019) with areas classified across multiple vegetation types including grass, forest and managed vegetation based on Sentinel-2 satellite data, was utilised to signify the availability of natural breeding sites and nectar availability for mosquito males. The percentage cover of each vegetation type was calculated within each sector as mosquitoes often show preferential areas to breed and rest (USGS, 2023a) Similarly, the averaged Landsat Normalized Difference Vegetation Index per sector was also utilised for this purpose (USGS, 2023b).

2.2 Residential Data

To represent both host density and urban breeding habitat availability, data on the locations of public housing estates named Housing Development Board (HDBs) where over 80% of Singapore's resident population reside was obtained from Onemap (OneMap, 2023). Utilising the HDB location and HDB resale data, the average age of HDB buildings was collected as older age is a well-established risk factor for higher dengue incidence (Sun et. al, 2023.) This is due to building deterioration providing additional breeding habitats in cracks and design features such as laundry poles which are no longer built due to the pooling of water within the supports. Building height, which has also been correlated to dengue incidence, was calculated according to the number of floors and average height of each level of 3m. The number of condominiums and landed properties was additionally collected within each sector representing additional hosts being available. The percentage cover of built area was calculated as a sum of all residential, commercial and industrial buildings, representing the level of urbanicity, which has been associated with *Ae. aegpyti* presence (Kolimenakis et al., 2021) The major open drainage network for Singapore was obtained from the Public Utilities Board as a key breeding site for mosquitoes around HDBs. The average distance of each HDB block within a sector to a drain was measured as well as the length of the network within the sector (Fernandez et al., 2023)

2.3 Meteorological Data

For meteorological data, well-established variables which are known to affect mosquito survival or fecundity were collected. These included daily mean temperature, total rainfall, maximum rainfall falling within a 60-minute window, and wind speed, which were obtained from a total of 21 weather stations installed by the National Environment Agency. We created daily complete raster maps through inverse distance weighting interpolation, which was carried out using cross validation of leave-one-out for the fitting of the inverse distancing power to

minimise the error in observation on the raster surface of the test point. Ground air temperature was taken from ERA5, published by ECMWF (ECMWF, 2023). These values were aggregated at an annual level to correspond with the dengue case data (DoS, 2023)

2.4 Air Pollutant Data

We obtained daily air pollution data for a range of pollutants covering the years 2014−2020 from the Air Quality Open Data Platform. For Singapore, estimates are provided for 5 zones covering North, West, East, Central and South, covering PM_{10}, SO_2, NO_2, CO, and O_3. A fishnet grid of 650 by 650 points was then constructed resulting in a distance of approximately 120m between each point. Non-parametric missing value imputation using random Forest via the package *missForest* was conducted to downscale the coarse estimates to a finer grid. The methodology yields an out-of-bag imputation error estimate without the need of a test set or elaborate cross-validation. The average, minimum and/or maximum was calculated for these estimates within each sector

The estimates within each sector were then converted from AQI index levels to their respective concentration units using the breakpoints and formulas provided in the technical assistance documentation from the United States Environmental Protection Agency (US EPA, 2018), for use in the statistical models.

3. Analysis of Dengue Incidence Rates between 2014 – 2020

3.1 Spatial Autocorrelation

Table S1 : Moran's I Test Results of Dengue Incidence Rates of Public Housing Study Setting (2014-2020)

Year	Moran I Statistic	Expectation	Variance	Standard Deviate	P-Value	Observations
2014	0.36	-0.001	0.001	12.0	1.12e-33	849
2015	0.17	-0.001	0.001	5.87	2.18e-09	849
2016	0.10	-0.001	0.001	3.36	3.86e-04	849
2017	0.01	-0.001	0.001	0.55	0.29	849
2018	0.07	-0.001	0.001	2.18	0.01	750
2019	0.00	-0.001	0.001	0.10	0.46	750
2020	0.31	-0.001	0.001	9.12	3.76e-20	715

Table S1 *Moran's I results of the distribution of dengue incidence rates among the public housing spatial units. Observations vary through the study period as spatial units where Wolbachia intervention was carried out were excluded from the study. Expectation and Variance correspond to Moran I's statistic under the assumption of the null hypothesis that there is no spatial autocorrelation.*

Table S2 : Moran's I Test Results of Dengue Incidence Rates of Private Housing Study Setting (2014-2020)

Year	Moran I Statistic	Expectation	Variance	Standard Deviate	P-Value	Observations
2014	0.06	-0.003	0.001	1.73	0.04	404
2015	0.23	-0.003	0.002	5.11	1.59e-07	404
2016	0.18	-0.003	0.002	4.09	2.14e-05	404
2017	0.002	-0.003	0.001	0.17	0.43	404
2018	0.02	-0.003	0.0003	1.12	0.13	404
2019	0.33	-0.003	0.002	7.17	3.88e-13	404
2020	0.29	-0.003	0.002	6.21	2.64e-10	404

Table S2 *Moran's I results of the distribution of dengue incidence rates among the private housing spatial units. Expectation and Variance correspond to Moran I's statistic under the assumption of the null hypothesis that there is no spatial autocorrelation.*

Table S3: Moran's I Test Results of Model Residuals of Public Housing Study Setting (2014-2020)

Year	Moran I Statistic	Expectation	Variance	Standard Deviate	P-Value	Observations
2014	-0.02	-0.001	0.001	-0.72	0.76	849
2015	-0.02	-0.001	0.001	-0.49	0.69	849
2016	0.02	-0.001	0.001	0.69	0.24	849
2017	0.02	-0.001	0.001	0.80	0.21	849
2018	0.03	-0.001	0.001	1.02	0.15	750
2019	0.01	-0.001	0.001	0.28	0.39	750
2020	0.00	-0.001	0.001	-0.06	0.52	715

Table S3 *Moran's I results of the model residuals among the public housing spatial units. Observations vary through the study period as spatial units where Wolbachia intervention was carried out were excluded from the study. Expectation and Variance correspond to Moran I's statistic under the assumption of the null hypothesis that there is no spatial autocorrelation.*

Table S4: Moran's I Test Results of Model Residuals of Private Housing Study Setting (2014-2020)

Year	Moran I Statistic	Expectation	Variance	Standard Deviate	P-Value	Observations
2014	-0.05	-0.003	0.002	-0.91	0.82	404
2015	-0.05	-0.003	0.002	-1.03	0.85	404
2016	0.02	-0.003	0.002	0.39	0.35	404
2017	-0.01	-0.003	0.002	-0.13	0.55	404
2018	-0.01	-0.003	0.002	-0.08	0.53	404
2019	-0.09	-0.003	0.002	-1.82	0.97	404
2020	-0.05	-0.003	0.002	-1.02	0.85	404

Table S4 *Moran's I results of the model residuals among the private housing spatial units. Expectation and Variance correspond to Moran I's statistic under the assumption of the null hypothesis that there is no spatial autocorrelation.*

3.2 Summary Statistics

Table S5: Summary Statistics of Public Housing Study Setting (2014-2020)

Statistic	Mean	St. Dev.	Min	Pctl(25)	Median	Pctl(75)	Max
Current Year Incidence Rate (Cases per 1,000 person-years)	1.57	4.54	0.00	0.00	0.48	1.31	119.05
Population	5,103.63	2,661.30	216	3,216	4,680	6,504	20,492
Preceding Year Incidence Rate (Cases per 1,000 person-years)	1.45	4.41	0.00	0.00	0.46	1.21	119.05
NDVI	0.32	0.06	0.05	0.29	0.32	0.36	0.50
Total Vegetation Area (%)	0.04	0.08	0.00	0.00	0.00	0.05	0.63
Forest Cover (%)	0.001	0.01	0.00	0.00	0.00	0.00	0.20
Grass Cover (%)	0.01	0.04	0.00	0.00	0.00	0.00	0.39
Managed Vegetation Cover (%)	0.02	0.05	0.00	0.00	0.00	0.02	0.62
Building Area (%)	0.25	0.07	0.05	0.21	0.25	0.28	0.65
Number of Public Housing Units	609.77	719.96	0	0	404	1,099	4,374
Average Public Housing Building Height (m)	37.56	12.32	6.50	30.75	36.00	42.00	150.00
Average Public Housing Building Age (years)	29.10	11.98	0.00	22.83	30.53	37.50	85.00
Distance of Centroid to Drainage Network (m)	407.20	289.54	2.31	174.13	344.60	554.51	1,742.19
Length of Drainage Network in Spatial Unit (m)	50.47	130.61	0.00	0.00	0.00	0.00	1,113.12
Area within 300m of a Waterbody (%)	0.33	0.40	0.00	0.00	0.05	0.71	1.00
Area within 500m of a Waterbody (%)	0.57	0.44	0.00	0.00	0.75	1.00	1.00
Total Daily Rainfall (mm)	6.13	1.16	3.26	5.24	6.14	7.14	9.08
Highest 60-Minute Rainfall (mm)	4.68	0.88	2.44	4.04	4.68	5.31	6.90
Mean Temperature (°C)	28.04	0.26	27.45	27.85	28.01	28.19	28.88
Mean Wind Speed (km/h)	8.47	1.20	4.53	7.61	8.33	9.32	13.08
Mean Annual PM_{10} Concentration ($\mu g/m^3$)	29.27	4.46	21.04	26.24	28.09	32.02	39.38
Mean Annual O_3 Concentration (ppm)	0.02	0.002	0.01	0.02	0.02	0.02	0.02
Mean annual NO_2 Concentration (ppb)	13.81	4.60	6.55	10.70	13.35	15.90	27.56
Mean annual SO_2 Concentration (ppb)	3.48	1.34	1.29	2.47	3.39	4.51	7.93
Mean annual CO Concentration (ppm)	0.39	0.09	0.23	0.35	0.36	0.44	0.65

Table S5 *Summary statistics of the variables in the public housing spatial units (n = 5,611), for the study period of 2014 to 2020.*

Table S6: Summary Statistics of Private Housing Study Setting (2014-2020)

Statistic	Mean	St. Dev.	Min	Pctl(25)	Median	Pctl(75)	Max
Current Year Incidence Rate (Cases per 1,000 person-years)	7.43	19.57	0.00	0.00	0.00	6.54	333.33
Population	949.00	852.25	12	432	712.5	1,173	8,340
Preceding Year Incidence Rate (Cases per 1,000 person-years)	5.86	18.11	0.00	0.00	0.00	4.89	333.33
NDVI	0.32	0.06	0.11	0.27	0.32	0.37	0.49
Total Vegetation Area (%)	0.04	0.09	0.00	0.00	0.01	0.05	0.87
Forest Cover (%)	0.01	0.07	0.00	0.00	0.00	0.00	0.81
Grass Cover (%)	0.01	0.03	0.00	0.00	0.00	0.00	0.32
Managed Vegetation Cover (%)	0.02	0.03	0.00	0.00	0.00	0.02	0.28
Building Area (%)	0.32	0.09	0.00	0.28	0.33	0.38	0.64
Number of Condominium Units	10.00	19.43	0	0	2	13	243
Number of Landed Housing Units	161.67	122.21	0	73	140	218.2	702
Number of Public Housing Units	1,229.55	677.14	0	747	1,175	1,630.8	3,950
Distance of Centroid to Drainage Network (m)	325.61	226.01	1.68	156.02	282.38	442.42	1,382.79
Length of Drainage Network in Spatial Unit (m)	103.26	237.07	0.00	0.00	0.00	112.98	1,636.08
Area within 300m of a Waterbody (%)	0.25	0.36	0.00	0.00	0.00	0.50	1.00
Area within 500m of a Waterbody (%)	0.46	0.44	0.00	0.00	0.44	1.00	1.00
Total Daily Rainfall (mm)	5.85	1.27	3.16	4.88	5.80	6.93	9.09
Highest 60-Minute Rainfall (mm)	4.44	0.95	2.47	3.73	4.50	5.18	6.89
Mean Temperature (°C)	28.11	0.26	27.47	27.92	28.12	28.25	28.85
Mean Wind Speed (km/h)	8.68	1.10	4.82	7.94	8.63	9.41	13.36
Mean Annual PM_{10} Concentration ($\mu g/m^3$)	29.30	4.50	21.04	26.55	29.06	32.02	39.38
Mean Annual O_3 Concentration (ppm)	0.02	0.002	0.01	0.02	0.02	0.02	0.02
Mean annual NO_2 Concentration (ppb)	15.02	5.33	6.55	10.95	14.50	16.21	27.56
Mean annual SO_2 Concentration (ppb)	3.41	1.22	1.29	2.47	3.48	3.97	7.93
Mean annual CO Concentration (ppm)	0.39	0.10	0.23	0.35	0.35	0.44	0.65

Table S6 *Summary statistics of variables included in private housing spatial units (n = 2,828), for the study period of 2014 to 2020.*

3.2 Sensitivity Analysis

Table S7: Sensitivity Analysis of Public Housing Study Setting (2014-2020)

			Dependent variable:				
			Annual Dengue Incidence Rate (Cases per 1,000 person-years)				
	(1)	(2)	(3)	(4)	(5)	(6)	(7)
Preceding Year Incidence Rate (Cases per 1,000 person-years)	✓	✓	✓	✓	✓	✓	✓
Year	✓	✓	✓	✓	✓	✓	✓
NDVI	✓	✓	✓	✓	✓	✓	✓
Total Vegetation Area (%)	✓	✓	✓	✓	✓		
Forest Cover (%)	✓	✓	✓	✓	✓	✓	✓
Grass Cover (%)	✓	✓					
Managed Vegetation Cover (%)	✓	✓	✓	✓	✓	✓	✓
Building Area (%)	✓	✓	✓	✓	✓	✓	✓
Number of Public Housing Units	✓	✓	✓	✓	✓	✓	✓
Average Public Housing Building Height (m)	✓	✓	✓	✓	✓	✓	✓
Average Public Housing Building Age (years)	✓	✓	✓	✓	✓	✓	✓
Distance of Centroid to Drainage Network (m)	✓	✓	✓	✓	✓	✓	✓
Length of Drainage Network in Spatial Unit (m)	✓	✓	✓	✓	✓	✓	
Area Within 300m of a Waterbody (%)	✓						

Area Within 500m of a Waterbody (%)		✓	✓					
Total Daily Rainfall (mm)		✓	✓	✓	✓	✓	✓	✓
Highest 60-Minute Rainfall (mm)		✓	✓	✓	✓	✓	✓	✓
Mean Temperature (°C)		✓	✓	✓	✓	✓	✓	✓
Mean Wind Speed (km/h)		✓	✓	✓	✓	✓	✓	✓
Mean Annual PM_{10} Concentration (µg/m^3)		✓	✓	✓	✓	✓	✓	✓
Mean Annual O_3 Concentration (ppm)		✓	✓	✓	✓	✓	✓	✓
Mean annual NO_2 concentration (ppb)		✓	✓	✓	✓	✓	✓	✓
Mean annual SO_2 concentration (ppb)		✓	✓	✓	✓	✓	✓	✓
Mean annual CO concentration (ppm)		✓	✓	✓				
GLM	AIC	29,545	29,543	29,541	29,539	29,539	29,537	29,537
	Deviance Explained	16.7%	16.7%	16.7%	16.7%	16.7%	16.7%	16.7%
GAM	AIC	28,507	28,508	28,480	28,489	28,474	28,513	28,494
	Deviance Explained	32.6%	32.5%	33.0%	32.6%	33%	32.3%	32.6%

Table S7 *Summary of sensitivity analysis conducted in public housing spatial units (n = 5,611. Ticks indicate that the corresponding exposure was included in each respective Generalized Additive Model (GAM) or Generalized Linear Model (GLM). Exposures were removed one at a time using the backward stepwise regression method. Deviance explained indicates the percentage difference between the deviance of a model with exposures added and the model's null deviance.*

Table S8: Sensitivity Analysis of Private Housing Study Setting (2014-2020)

	Dependent variable:								
	Annual Dengue Incidence Rate (Cases per 1,000 person-years)								
	(1)	(2)	(3)	(4)	(5)	(6)	(7)	(8)	(9)
Preceding Year Incidence Rate (Cases per 1,000 person-years)	✓	✓	✓	✓	✓	✓	✓	✓	✓
Year	✓	✓	✓	✓	✓	✓	✓	✓	✓
NDVI	✓	✓							
Total Vegetation Area (%)	✓	✓	✓	✓	✓	✓			
Forest Cover (%)	✓	✓	✓	✓	✓				
Grass Cover (%)	✓	✓	✓	✓	✓	✓	✓	✓	✓
Manged Vegetation Cover (%)	✓	✓	✓	✓	✓	✓	✓	✓	✓
Building Area (%)	✓	✓	✓	✓	✓	✓	✓	✓	✓
Number of Condominium Units	✓	✓	✓	✓	✓	✓	✓		
Number of Landed Housing Units	✓	✓	✓	✓	✓	✓	✓	✓	✓
Number of Public Housing Units	✓								
Distance of Centroid to Drainage Network (m)	✓	✓	✓	✓	✓	✓	✓	✓	
Length of Drainage Network in Spatial Unit (m)	✓	✓	✓	✓	✓	✓	✓	✓	✓
Area Within 300m of a Waterbody (%)	✓	✓	✓	✓	✓	✓	✓	✓	✓
Area Within 500m of a Waterbody (%)	✓	✓	✓	✓					
Total Daily Rainfall (mm)	✓	✓	✓	✓	✓	✓	✓	✓	✓
Highest 60-Minute Rainfall (mm)	✓	✓	✓	✓	✓	✓	✓	✓	✓

Mean Temperature (°C)		✓	✓	✓	✓	✓	✓	✓	✓	✓
Mean Wind Speed (km/h)		✓	✓	✓	✓	✓	✓	✓	✓	✓
Mean Annual PM_{10} Concentration ($\mu g/m^3$)		✓	✓	✓						
Mean Annual O_3 Concentration (ppm)		✓	✓	✓	✓	✓	✓	✓	✓	✓
Mean annual NO_2 concentration (ppb)		✓	✓	✓	✓	✓	✓	✓	✓	✓
Mean annual SO_2 concentration (ppb)		✓	✓	✓	✓	✓	✓	✓	✓	✓
Mean annual CO concentration (ppm)		✓	✓	✓	✓	✓	✓	✓	✓	✓
GLM	AIC	12,510	12,508	12,506	12,504	12,502	12,501	12,499	12,497	12,496
	Deviance Explained	20.0%	20.0%	20.0%	20.0%	20.0%	20.0%	20.0%	20.0%	19.9%
GAM	AIC	11,939	11,937	11,940	11,940	11,941	11,941	11,940	11,948	11,946
	Deviance Explained	40.5%	40.2%	39.8%	39.9%	39.7%	39.7%	39.7%	39.3%	39.3%

Table S8 *Summary of sensitivity analysis conducted for the private housing spatial units (n = 2,828. Ticks indicate that the corresponding exposure was included in each respective Generalized Additive Model (GAM) or Generalized Linear Model (GLM). Exposures were removed one at a time using the backward stepwise regression method. Deviance explained indicates the percentage difference between the deviance of a model with exposures added and the model's null deviance.*

3.3 Regression Outputs from Linear Models

Table S9: Regression Results of Public Housing Study Setting (2014-2020)

	(1)	(2)	(3)	(4)	(5)	(6)	(7)
(Intercept)	0.00 ***	0.00 ***	0.00 ***	0.00 ***	0.00 ***	0.00 ***	0.00 ***
	[0.00, 0.00]	[0.00, 0.00]	[0.00, 0.00]	[0.00, 0.00]	[0.00, 0.00]	[0.00, 0.00]	[0.00, 0.00]
Preceding Year Incidence Rate	1.02 ***	1.02 ***	1.02 ***	1.02 ***	1.02 ***	1.02 ***	1.02 ***
(Cases per 1,000 person-years)	[1.01, 1.03]	[1.01, 1.03]	[1.01, 1.03]	[1.01, 1.03]	[1.01, 1.03]	[1.01, 1.03]	[1.01, 1.03]
Year	1.13 ***	1.13 ***	1.13 ***	1.13 ***	1.14 ***	1.14 ***	1.14 ***
	[1.08, 1.18]	[1.08, 1.18]	[1.08, 1.18]	[1.08, 1.18]	[1.10, 1.18]	[1.10, 1.18]	[1.10, 1.18]
NDVI	0.43 *	0.43 *	0.43 *	0.43 *	0.43 *	0.44	0.45
	[0.19, 0.97]	[0.19, 0.97]	[0.19, 0.97]	[0.19, 0.97]	[0.19, 0.98]	[0.20, 1.01]	[0.20, 1.03]
Total Vegetation Area(%)	1.55	1.55	1.55	1.50	1.48		
	[0.69, 3.50]	[0.69, 3.50]	[0.69, 3.50]	[0.75, 3.00]	[0.74, 2.97]		
Forest Cover (%)	0.02 *	0.02 *	0.02 *	0.02 *	0.02 *	0.02 *	0.02 *
	[0.00, 0.43]	[0.00, 0.43]	[0.00, 0.43]	[0.00, 0.43]	[0.00, 0.43]	[0.00, 0.46]	[0.00, 0.45]
Grass Cover (%)	0.89	0.89	0.88				
	[0.25, 3.18]	[0.25, 3.18]	[0.25, 3.14]				
Managed Vegetation Cover (%)	2.20	2.20	2.21	2.24	2.28	3.41 **	3.44 **
	[0.70, 6.86]	[0.70, 6.86]	[0.71, 6.88]	[0.74, 6.76]	[0.75, 6.88]	[1.45, 8.00]	[1.47, 8.07]
Building Area (%)	3.49 ***	3.49 ***	3.50 ***	3.52 ***	3.50 ***	3.55 ***	3.32 ***
	[1.72, 7.07]	[1.72, 7.07]	[1.73, 7.10]	[1.74, 7.12]	[1.73, 7.07]	[1.76, 7.18]	[1.65, 6.68]
Number of Public Housing Units	1.00	1.00	1.00	1.00	1.00	1.00	1.00
	[1.00, 1.00]	[1.00, 1.00]	[1.00, 1.00]	[1.00, 1.00]	[1.00, 1.00]	[1.00, 1.00]	[1.00, 1.00]
Average Public Housing Building Height (m)	0.99 ***	0.99 ***	0.99 ***	0.99 ***	0.99 ***	0.99 ***	0.99 ***
	[0.99, 0.99]	[0.99, 0.99]	[0.99, 0.99]	[0.99, 0.99]	[0.99, 0.99]	[0.98, 0.99]	[0.98, 0.99]
Average Public Housing Building Age (years)	1.03 ***	1.03 ***	1.03 ***	1.03 ***	1.03 ***	1.03 ***	1.03 ***
	[1.02, 1.03]	[1.02, 1.03]	[1.02, 1.03]	[1.02, 1.03]	[1.02, 1.03]	[1.02, 1.03]	[1.02, 1.03]

Distance of Centroid to Drainage Network (m)	1.00	1.00	1.00	1.00	1.00	1.00	1.00
	[1.00, 1.00]	[1.00, 1.00]	[1.00, 1.00]	[1.00, 1.00]	[1.00, 1.00]	[1.00, 1.00]	[1.00, 1.00]
Length of Drainage Network in Spatial Unit (m)	1.00	1.00	1.00	1.00	1.00	1.00	
	[1.00, 1.00]	[1.00, 1.00]	[1.00, 1.00]	[1.00, 1.00]	[1.00, 1.00]	[1.00, 1.00]	
Area Within 300m of a Waterbody (%)	1.00						
	[0.84, 1.19]						
Area Within 500m of a Waterbody (%)	0.99	0.99					
	[0.85, 1.17]	[0.90, 1.10]					
Total Daily Rainfall (mm)	0.48 ***	0.48 ***	0.48 ***	0.48 ***	0.48 ***	0.48 ***	0.48 ***
	[0.38, 0.61]	[0.38, 0.61]	[0.38, 0.61]	[0.38, 0.61]	[0.38, 0.61]	[0.38, 0.60]	[0.38, 0.61]
Highest 60-Minute Rainfall (mm)	2.10 ***	2.10 ***	2.10 ***	2.10 ***	2.08 ***	2.09 ***	2.08 ***
	[1.54, 2.88]	[1.54, 2.88]	[1.54, 2.88]	[1.54, 2.88]	[1.53, 2.84]	[1.54, 2.86]	[1.52, 2.83]
Mean Temperature (°C)	6.40 ***	6.40 ***	6.40 ***	6.40 ***	6.24 ***	6.27 ***	6.25 ***
	[4.91, 8.34]	[4.91, 8.34]	[4.91, 8.34]	[4.91, 8.34]	[4.83, 8.07]	[4.86, 8.11]	[4.84, 8.07]
Mean Wind Speed (km/h)	0.96	0.96	0.96	0.96	0.95	0.95	0.95
	[0.90, 1.01]	[0.90, 1.01]	[0.90, 1.01]	[0.90, 1.01]	[0.90, 1.00]	[0.90, 1.00]	[0.90, 1.00]
Mean Annual PM_{10} Concentration (mg/m^3)	0.95 ***	0.95 ***	0.95 ***	0.95 ***	0.96 ***	0.96 ***	0.96 ***
	[0.94, 0.97]	[0.94, 0.97]	[0.94, 0.97]	[0.94, 0.97]	[0.94, 0.97]	[0.94, 0.97]	[0.94, 0.97]
Mean Annual O_3 Concentration (ppm)	0.00 **	0.00 **	0.00 **	0.00 **	0.00 **	0.00 **	0.00 **
	[0.00, 0.00]	[0.00, 0.00]	[0.00, 0.00]	[0.00, 0.00]	[0.00, 0.00]	[0.00, 0.00]	[0.00, 0.00]
Mean annual NO_2 concentration (ppb)	1.04 ***	1.04 ***	1.04 ***	1.04 ***	1.04 ***	1.04 ***	1.04 ***
	[1.03, 1.06]	[1.03, 1.06]	[1.03, 1.06]	[1.03, 1.06]	[1.03, 1.06]	[1.03, 1.06]	[1.03, 1.06]
Mean annual SO_2 concentration (ppb)	0.95 *	0.95 *	0.95 *	0.95 *	0.95 *	0.95 *	0.95 **
	[0.91, 0.99]	[0.91, 0.99]	[0.91, 0.99]	[0.91, 0.99]	[0.91, 0.99]	[0.91, 0.99]	[0.91, 0.99]
Mean annual CO concentration (ppm)	1.30	1.30	1.30	1.30			

	[0.65, 2.61]	[0.65, 2.61]	[0.64, 2.60]	[0.65, 2.61]			
AIC	29,545	29,543	29,541	29,539	29,539	29,537	29,537

*** p < 0.001; ** p < 0.01; * p < 0.05

Table S9 *Regression results from Generalized Linear Models for public housing spatial units (n =5,611), containing data from 2014 to 2020. Coefficients presented in this table have been exponentiated to obtain Incidence Rate Ratios (IRRs), for easier interpretation of results. Model (4), containing all exposures considered in this study, demonstrated best fit, with the lowest AIC in contrast to remaining models. Values in brackets represent 95% confidence intervals.*

Table S10: Regression Results of Private Housing Study Setting (2014 - 2020)

	(1)	(2)	(3)	(4)	(5)	(6)	(7)	(8)	(9)
(Intercept)	0*** [0.00,0.00]	0*** [0.00,0.00]	0*** [0.00,0.00]	0*** [0.00,0.00]	0*** [0.00,0.00]	0*** [0.00,0.00]	0*** [0.00,0.00]	0*** [0.00,0.00]	0*** [0.00,0.00]
Preceding Year Incidence Rate (Cases per 1,000 person-years)	1.01 *** [1.01, 1.02]	1.01 *** [1.01, 1.02]	1.01 *** [1.01, 1.02]	1.01 *** [1.01, 1.02]	1.01 *** [1.01, 1.02]	1.01 *** [1.01, 1.02]	1.01 *** [1.01, 1.02]	1.01 *** [1.01, 1.02]	1.01 *** [1.01, 1.02]
Year	0.83 *** [0.76, 0.91]	0.83 *** [0.76, 0.91]	0.83 *** [0.76, 0.91]	0.83 *** [0.77, 0.91]	0.83 *** [0.76, 0.91]	0.83 *** [0.77, 0.91]	0.83 *** [0.77, 0.91]	0.83 *** [0.77, 0.91]	0.84 *** [0.77, 0.91]
NDVI	1.17 [0.30, 4.56]	1.18 [0.30, 4.57]							
Total Vegetation Area (%)	0.31 [0.01, 6.27]	0.31 [0.01, 6.24]	0.31 [0.02, 6.30]	0.31 [0.02, 6.37]	0.32 [0.02, 6.50]	0.84 [0.26, 2.70]			
Forest Cover (%)	3.15 [0.11, 92.43]	3.16 [0.11, 92.63]	3.17 [0.11, 92.96]	3.10 [0.11, 90.78]	3.00 [0.10, 87.73]				
Grass Cover (%)	212.91 ** [4.29, 10566.71]	217.76 ** [4.39, 10801.43]	223.60 ** [4.51, 11076.53]	215.34 ** [4.35, 10669.84]	180.34 ** [3.75, 8683.08]	78.07 ** [3.60, 1691.53]	67.67 ** [3.84, 1192.43]	71.63 ** [4.07, 1259.27]	68.05 ** [3.86, 1201.05]
Managed Vegetation Cover (%)	56.02 * [1.38, 2272.35]	55.23 * [1.37, 2228.82]	56.95 * [1.43, 2261.76]	54.41 * [1.37, 2160.22]	58.72 * [1.49, 2307.34]	23.75 * [1.82, 310.28]	21.10 * [1.88, 237.17]	21.26 * [1.90, 238.36]	19.73 * [1.76, 220.82]
Building Area (%)	3.59 * [1.23, 10.47]	3.58 * [1.23, 10.44]	3.44 * [1.22, 9.72]	3.37 * [1.20, 9.46]	3.42 * [1.22, 9.60]	3.28 * [1.18, 9.11]	3.49 * [1.32, 9.24]	3.56 * [1.35, 9.41]	3.38 * [1.29, 8.86]
Number of Condominium Units	1.00 [1.00, 1.00]	1.00 [1.00, 1.00]	1.00 [1.00, 1.00]	1.00 [1.00, 1.00]	1.00 [1.00, 1.00]	1.00 [1.00, 1.00]	1.00 [1.00, 1.00]		
Number of Landed Units	1.00 [1.00, 1.00]	1.00 [1.00, 1.00]	1.00 [1.00, 1.00]	1.00 [1.00, 1.00]	1.00 [1.00, 1.00]	1.00 [1.00, 1.00]	1.00 [1.00, 1.00]	1.00 [1.00, 1.00]	1.00 [1.00, 1.00]
Number of Public Housing Units	1.00 [1.00, 1.00]								

Distance of Centroid to Drainage Network (m)	1.00 [1.00, 1.00]	1.00 [1.00, 1.00]	1.00 [1.00, 1.00]	1.00 [1.00, 1.00]	1.00 [1.00, 1.00]	1.00 [1.00, 1.00]	1.00 [1.00, 1.00]	1.00 [1.00, 1.00]	
Length of Drainage Network in Spatial Unit (m)	1.00 [1.00, 1.00]	1.00 [1.00, 1.00]	1.00 [1.00, 1.00]	1.00 [1.00, 1.00]	1.00 [1.00, 1.00]	1.00 [1.00, 1.00]	1.00 [1.00, 1.00]	1.00 [1.00, 1.00]	1.00 [1.00, 1.00]
Area Within 300m of a Waterbody (%)	1.60 ** [1.12, 2.28]	1.60 ** [1.12, 2.29]	1.59 ** [1.12, 2.25]	1.60 ** [1.13, 2.26]	1.48 *** [1.20, 1.83]	1.47 *** [1.19, 1.82]	1.47 *** [1.19, 1.82]	1.48 *** [1.19, 1.82]	1.44 *** [1.17, 1.77]
Area Within 500m of a Waterbody (%)	0.92 [0.68, 1.23]	0.91 [0.68, 1.23]	0.92 [0.69, 1.23]	0.92 [0.69, 1.23]					
Total Daily Rainfall (mm)	0.46 *** [0.29, 0.71]	0.46 *** [0.29, 0.71]	0.46 *** [0.29, 0.71]	0.44 *** [0.29, 0.68]	0.44 *** [0.29, 0.67]	0.44 *** [0.28, 0.67]	0.43 *** [0.28, 0.67]	0.43 *** [0.28, 0.66]	0.43 *** [0.28, 0.66]
Highest 60-Minute Rainfall (mm)	3.58 *** [1.99, 6.43]	3.57 *** [1.99, 6.42]	3.57 *** [1.99, 6.41]	3.69 *** [2.07, 6.59]	3.76 *** [2.11, 6.71]	3.77 *** [2.11, 6.73]	3.80 *** [2.13, 6.77]	3.82 *** [2.14, 6.80]	3.81 *** [2.14, 6.80]
Mean Temperature (°C)	7.78 *** [4.93, 12.28]	7.80 *** [4.94, 12.32]	7.79 *** [4.94, 12.30]	7.69 *** [4.91, 12.05]	7.74 *** [4.94, 12.12]	7.73 *** [4.94, 12.10]	7.70 *** [4.92, 12.04]	7.71 *** [4.93, 12.06]	7.63 *** [4.88, 11.92]
Mean Wind Speed (km/h)	1.55 *** [1.39, 1.72]	1.55 *** [1.39, 1.72]	1.55 *** [1.39, 1.72]	1.55 *** [1.39, 1.72]	1.56 *** [1.40, 1.73]	1.55 *** [1.40, 1.72]	1.55 *** [1.40, 1.73]	1.55 *** [1.40, 1.72]	1.54 *** [1.39, 1.71]
Mean Annual PM_{10} Concentration (mg/m^3)	1.01 [0.98, 1.03]	1.01 [0.98, 1.03]	1.01 [0.98, 1.03]						
Mean Annual O_3 Concentration (ppm)	0.00 *** [0.00, 0.00]	0.00 *** [0.00, 0.00]	0.00 *** [0.00, 0.00]	0.00 *** [0.00, 0.00]	0.00 *** [0.00, 0.00]	0.00 *** [0.00, 0.00]	0.00 *** [0.00, 0.00]	0.00 *** [0.00, 0.00]	0.00 *** [0.00, 0.00]
Mean annual NO_2 concentration (ppb)	1.09 *** [1.06, 1.11]	1.09 *** [1.06, 1.11]	1.09 *** [1.06, 1.11]	1.09 *** [1.06, 1.11]	1.09 *** [1.07, 1.11]	1.09 *** [1.07, 1.11]	1.09 *** [1.07, 1.11]	1.09 *** [1.07, 1.11]	1.09 *** [1.07, 1.11]
Mean annual SO_2 concentration (ppb)	0.81 *** [0.74, 0.89]	0.81 *** [0.74, 0.89]	0.81 *** [0.74, 0.89]	0.82 *** [0.75, 0.90]	0.82 *** [0.75, 0.90]	0.82 *** [0.75, 0.90]	0.82 *** [0.75, 0.90]	0.82 *** [0.75, 0.90]	0.82 *** [0.75, 0.90]
Mean annual CO concentration (ppm)	7.25 *** [2.46, 21.38]	7.25 *** [2.46, 21.37]	7.19 *** [2.44, 21.19]	7.98 *** [2.84, 22.42]	8.29 *** [2.95, 23.30]	8.35 *** [2.97, 23.45]	8.37 *** [2.98, 23.52]	8.45 *** [3.01, 23.71]	8.10 *** [2.89, 22.74]
AIC	12,510	12,508	12,506	12,504	12,502	12,501	12,499	12,497	12,496

*** $p < 0.001$; ** $p < 0.01$; * $p < 0.05$.

Table S10 *Regression results from Generalized Linear Models for private housing spatial units (n =2,828), containing data from 2014 to 2020. Coefficients presented in this table have been exponentiated to obtain Incidence Rate Ratios (IRRs), for easier interpretation of results. Model (4), containing all exposures considered in this study, demonstrated best fit, with the lowest AIC in contrast to remaining models. Values in brackets represent 95% confidence intervals..*

4. Analysis of Dengue Incidence Rates from 2008 to 2020

4.1 Study Setting

A similar analysis as described in the main text was carried out on a dataset comprising of the same spatial units and a subset of the variables presented in our study from 2008 to 2020. The variables present in this dataset are described in Tables S7 and S8.

We first used Generalized Linear Models (GLMs) to model linear relationships between the exposures and dengue incidence rates. Following which, the assumption of linearity was relaxed and Generalized Additive Models were utilized to measure potential non-linear associations between the exposures and the outcome. Once again, we constructed four different sets of models for both GLMs and GAMS: (1) a model containing only vegetation exposures, (2) a model containing vegetation and anthropogenic exposures, (3) a model containing vegetation, anthropogenic, drainage and water source exposures and (4) a model containing all exposures present in the dataset. The comparison of model-fit was determined using the Akaike Information Criterion (AIC). The sensitivity analysis and regression outputs from the models are presented in the following sections.

While the models from these dataset contain more data, the results from the shorter study period from 2014 to 2020 were interpreted and presented. The study period between 2014 to 2020 allows us to study the combined effects of a greater amount of exposures, which outweighs the benefits provided by having a larger pool of data.

4.2 Summary Statistics

Table S11: Summary Statistics of Public Housing Study Setting (2008-2020)

Statistic	Mean	St. Dev.	Min	Pctl(25)	Median	Pctl(75)	Max
Current Year Incidence Rate (Cases per 1,000 person-years)	1.04	3.47	0.00	0.00	0.12	0.87	119.05
Population	5,112.28	2,667.08	216	3,220	4,692	6,504	20,492
Preceding Year Incidence Rate (Cases per 1,000 person-years)	0.86	3.29	0.00	0.00	0.00	0.69	119.05
NDVI	0.32	0.06	0.05	0.29	0.32	0.36	0.50
Vegetation Density	0.04	0.08	0.00	0.00	0.00	0.05	0.63
Forest Cover (%)	0.001	0.01	0.00	0.00	0.00	0.00	0.20
Grass Cover (%)	0.01	0.04	0.00	0.00	0.00	0.00	0.39
Total Vegetation Area (%)	0.02	0.05	0.00	0.00	0.00	0.02	0.62
Building Area (%)	0.25	0.07	0.05	0.21	0.25	0.28	0.65
Number of Public Housing Units	612.95	724.04	0	0	404	1,103	4,374
Average Public Housing Building Height (m)	37.40	12.24	6.50	30.63	36.00	41.63	150.00
Average Public Housing Building Age (years)	29.10	11.96	0.00	22.83	30.64	37.43	85.00
Distance of Centroid to Drainage Network (m)	408.41	289.26	2.31	174.65	346.69	559.32	1,742.19

Length of Drainage Network in Spatial Unit (m)	50.87	131.37	0.00	0.00	0.00	0.00	1,113.12
Area within 300m of a Waterbody (%)	0.32	0.40	0.00	0.00	0.05	0.70	1.00
Area within 500m of a Waterbody (%)	0.57	0.44	0.00	0.00	0.73	1.00	1.00
Total Daily Rainfall (mm)	6.73	1.25	3.26	5.79	6.96	7.60	9.88
Mean Temperature (°C)	27.82	0.34	27.04	27.63	27.81	28.04	28.88
Mean Wind Speed (km/h)	8.13	1.09	4.53	7.36	8.11	8.85	13.08

Table S11 *Summary statistics of the variables in the public housing spatial units(n = 10,705), during the study period of 2008 to 2020.*

Table S12: Summary Statistics of Private Housing Study Setting (2008-2020)

Statistic	Mean	St. Dev.	Min	Pctl(25)	Median	Pctl(75)	Max
Current Year Incidence Rate (Cases per 1,000 person-years)	4.87	15.49	0.00	0.00	0.00	3.30	333.33
Population	949.00	852.18	12	432	712.5	1,173	8,340
Preceding Year Incidence Rate (Cases per 1,000 person-years)	3.58	13.82	0.00	0.00	0.00	2.13	333.33
NDVI	0.32	0.06	0.11	0.27	0.32	0.37	0.49
Vegetation Density	0.04	0.09	0.00	0.00	0.01	0.05	0.87
Forest Cover (%)	0.01	0.07	0.00	0.00	0.00	0.00	0.81
Grass Cover (%)	0.01	0.03	0.00	0.00	0.00	0.00	0.32
Total Vegetation Area (%)	0.02	0.03	0.00	0.00	0.00	0.02	0.28
Building Area (%)	0.32	0.09	0.00	0.28	0.33	0.38	0.64
Number of Public Housing Units	1,229.55	677.09	0	747	1,175	1,630.8	3,950
Number of Condominium Units	10.00	19.43	0	0	2	13	243
Number of Landed Housing Units	161.67	122.20	0	73	140	218.2	702
Distance of Centroid to Drainage Network (m)	325.61	225.99	1.68	156.02	282.38	442.42	1,382.79
Length of Drainage Network in Spatial Unit (m)	103.26	237.05	0.00	0.00	0.00	112.98	1,636.08
Area within 300m of a Waterbody (%)	0.25	0.36	0.00	0.00	0.00	0.50	1.00
Area within 500m of a Waterbody (%)	0.46	0.44	0.00	0.00	0.44	1.00	1.00
Total Daily Rainfall (mm)	6.49	1.35	3.16	5.49	6.68	7.46	9.86
Mean Temperature (°C)	27.88	0.36	27.00	27.70	27.87	28.14	28.85
Mean Wind Speed (km/h)	8.27	1.08	4.82	7.55	8.16	8.93	13.36

Table S12 *Summary statistics of the variables in private housing spatial units (n = 5,252), during the study period of 2008 to 2020*

4.3 Sensitivity Analysis

Table S13: Sensitivity Analysis of Public Housing Study Setting (2008-2020)

				Dependent variable:			
				Annual Dengue Incidence Rate (Cases per 1,000 person-years)			
	(1)	(2)	(3)	(4)	(5)	(6)	(7)
Preceding Year Incidence Rate (Cases per 1,000 person-years)	✓	✓	✓	✓	✓	✓	✓
Year	✓	✓	✓	✓	✓	✓	✓
NDVI	✓	✓	✓	✓	✓	✓	
Total Vegetation Area (%)	✓	✓	✓	✓	✓	✓	✓
Forest Cover (%)	✓	✓	✓	✓	✓	✓	✓
Grass Cover (%)	✓	✓					
Managed Vegetation Cover (%)	✓	✓	✓	✓			
Building Area (%)	✓	✓	✓	✓	✓	✓	✓
Number of Public Housing Units	✓	✓	✓				
Average Public Housing Building Height (m)	✓	✓	✓	✓	✓	✓	✓
Average Public Housing Building Age (years)	✓	✓	✓	✓	✓	✓	✓
Distance of Centroid to Drainage Network (m)	✓						
Length of Drainage Network in Spatial Unit (m)	✓	✓	✓	✓	✓	✓	✓
Area Within 300m of a Waterbody (%)	✓	✓	✓	✓	✓		

Area Within 500m of a Waterbody (%)		✓	✓	✓	✓	✓	✓	✓
Total Daily Rainfall (mm)		✓	✓	✓	✓	✓	✓	✓
Mean Temperature (°C)		✓	✓	✓	✓	✓	✓	✓
Mean Wind Speed (km/h)		✓	✓	✓	✓	✓	✓	✓
GLM	AIC	45,359	45,357	45,355	45,354	45,352	45,351	45,351
	Deviance Explained	22.1%	22.1%	22.1%	22.1%	22.1%	22.0%	22.0%
GAM	AIC	43,485	43,472	43,471	43,476	43,475	43,483	43,487
	Deviance Explained	37.3%	37.5%	37.5%	37.4%	37.4%	37.3%	37.2%

Table S13 *Summary of sensitivity analysis conducted for public housing spatial units (n = 10,705) from the study period of 2008 to 2020. Ticks indicate that the corresponding exposure was included in each respective Generalized Additive Model (GAM) or Generalized Linear Model (GLM). Exposures were removed one at a time using the backward stepwise regression method. Deviance explained indicates the percentage difference between the deviance of a model with exposures added and the model's null deviance.*

Table S14: Sensitivity Analysis of Private Housing Study Setting (2008-2020)

	Dependent variable:				
	Annual Dengue Incidence Rate (Cases per 1,000 person-years)				
	(1)	(2)	(3)	(4)	(5)
Preceding Year Incidence Rate (Cases per 1,000 person-years)	✓	✓	✓	✓	✓
Year	✓	✓	✓	✓	✓
NDVI	✓	✓	✓	✓	✓
Total Vegetation Area (%)	✓	✓	✓		
Forest Cover (%)	✓	✓			
Grass Cover (%)	✓	✓	✓	✓	✓
Managed Vegetation Cover (%)	✓				

Building Area (%)	✓	✓	✓	✓	✓
Number of Public Housing Units	✓	✓	✓	✓	
Number of Condominium Units	✓	✓	✓	✓	✓
Number of Landed Housing Units	✓	✓	✓	✓	✓
Distance of Centroid to Drainage Network (m)	✓	✓	✓	✓	✓
Length of Drainage Network in Spatial Unit (m)	✓	✓	✓	✓	✓
Area Within 300m of a Waterbody (%)	✓	✓	✓	✓	✓
Area Within 500m of a Waterbody (%)	✓	✓	✓	✓	✓
Total Daily Rainfall (mm)	✓	✓	✓	✓	✓
Mean Temperature (°C)	✓	✓	✓	✓	✓
Mean Wind Speed (km/h)	✓	✓	✓	✓	✓
GLM AIC	18,702	18,700	18,699	18,697	18,697
GLM Deviance Explained	20.1%	20.1%	20.1%	20.1%	20.0%
GAM AIC	17,959	17,969	18,001	17,999	18,016
GAM Deviance Explained	36.7%	36.3%	35.6%	35.6%	35.0%

Table S14 *Summary of sensitivity analysis conducted for private housing spatial units (n = 5,252) from 2008 to 2020. Ticks indicate that the corresponding exposure was included in each respective Generalized Additive Model (GAM) or Generalized Linear Model (GLM). Exposures were removed one at a time using the backward stepwise regression method. Deviance explained indicates the percentage difference between the deviance of a model with exposures added and the model's null deviance.*

4.4 Regression Outputs from Linear Models

Table S15: Regression Results of Public Housing Study Setting (2008-2020)

	(1)	(2)	(3)	(4)	(5)	(6)	(7)
(Intercept)	0.00 ***<br>[0.00, 0.00]	0.00 ***<br>[0.00, 0.00]	0.00 ***<br>[0.00, 0.00]	0.00 ***<br>[0.00, 0.00]	0.00 ***<br>[0.00, 0.00]	0.00 ***<br>[0.00, 0.00]	0.00 ***<br>[0.00, 0.00]
Preceding Year Incidence Rate (Cases per 1,000 person-years)	1.01 *<br>[1.00, 1.02]	1.01 *<br>[1.00, 1.02]	1.01 *<br>[1.00, 1.02]	1.01 *<br>[1.00, 1.02]	1.01 *<br>[1.00, 1.02]	1.01 *<br>[1.00, 1.02]	1.01 *<br>[1.00, 1.02]
Year	1.18 ***<br>[1.17, 1.20]	1.18 ***<br>[1.17, 1.20]	1.18 ***<br>[1.17, 1.20]	1.19 ***<br>[1.17, 1.20]	1.19 ***<br>[1.17, 1.20]	1.18 ***<br>[1.17, 1.20]	1.19 ***<br>[1.17, 1.20]
NDVI	0.63<br>[0.31, 1.26]	0.63<br>[0.31, 1.26]	0.63<br>[0.31, 1.26]	0.62<br>[0.31, 1.24]	0.62<br>[0.31, 1.24]	0.61<br>[0.31, 1.23]	
Total Vegetation Area (%)	1.27<br>[0.63, 2.52]	1.30<br>[0.66, 2.57]	1.39<br>[0.77, 2.50]	1.39<br>[0.77, 2.51]	1.58 *<br>[1.01, 2.49]	1.58 *<br>[1.01, 2.48]	1.54<br>[0.98, 2.41]
Forest Cover (%)	0.00 ***<br>[0.00, 0.01]	0.00 ***<br>[0.00, 0.01]	0.00 ***<br>[0.00, 0.01]	0.00 ***<br>[0.00, 0.01]	0.00 ***<br>[0.00, 0.01]	0.00 ***<br>[0.00, 0.01]	0.00 ***<br>[0.00, 0.01]
Grass Cover (%)	1.29<br>[0.44, 3.78]	1.26<br>[0.43, 3.67]					
Managed Vegetation Cover (%)	1.49<br>[0.57, 3.93]	1.48<br>[0.56, 3.88]	1.43<br>[0.56, 3.67]	1.41<br>[0.55, 3.63]			
Building Area	1.78<br>[0.97, 3.27]	1.79<br>[0.98, 3.29]	1.78<br>[0.97, 3.25]	1.75<br>[0.96, 3.19]	1.69<br>[0.93, 3.09]	1.72<br>[0.94, 3.14]	1.94 *<br>[1.09, 3.46]
Number of Public Housing Units	1.00<br>[1.00, 1.00]	1.00<br>[1.00, 1.00]	1.00<br>[1.00, 1.00]				
Average Public Housing Building Height (m)	0.99 ***<br>[0.98, 0.99]	0.99 ***<br>[0.98, 0.99]	0.99 ***<br>[0.98, 0.99]	0.99 ***<br>[0.98, 0.99]	0.99 ***<br>[0.98, 0.99]	0.99 ***<br>[0.98, 0.99]	0.99 ***<br>[0.98, 0.99]
Average Public Housing Building Age (Years)	1.04 ***<br>[1.03, 1.04]	1.04 ***<br>[1.03, 1.04]	1.04 ***<br>[1.03, 1.04]	1.04 ***<br>[1.03, 1.04]	1.04 ***<br>[1.03, 1.04]	1.04 ***<br>[1.03, 1.04]	1.04 ***<br>[1.03, 1.04]
Distance of Centroid to Drainage Network (m)	1.00<br>[1.00, 1.00]						
Length of Drainage Network in Spatial Unit (m)	1.00<br>[1.00, 1.00]	1.00<br>[1.00, 1.00]	1.00<br>[1.00, 1.00]	1.00<br>[1.00, 1.00]	1.00<br>[1.00, 1.00]	1.00<br>[1.00, 1.00]	1.00<br>[1.00, 1.00]
Area Within 300m of a Waterbody (%)	0.93<br>[0.80, 1.08]	0.92<br>[0.80, 1.07]	0.92<br>[0.80, 1.07]	0.92<br>[0.80, 1.07]	0.92<br>[0.79, 1.07]		

Area Within 300m of a Waterbody (%)	0.86 * [0.76, 0.99]	0.86 * [0.76, 0.99]	0.87 * [0.76, 0.99]	0.87 * [0.76, 0.99]	0.87 * [0.76, 0.99]	0.82 *** [0.75, 0.89]	0.82 *** [0.75, 0.89]
Total Daily Rainfall (mm)	1.05 * [1.00, 1.09]	1.05 * [1.00, 1.09]	1.05 * [1.00, 1.09]	1.05 * [1.00, 1.09]	1.05 * [1.00, 1.09]	1.05 * [1.00, 1.09]	1.04 * [1.00, 1.09]
Mean Temperature (°C)	3.56 *** [2.97, 4.28]	3.54 *** [2.95, 4.25]	3.55 *** [2.96, 4.26]	3.53 *** [2.95, 4.24]	3.53 *** [2.94, 4.23]	3.53 *** [2.95, 4.23]	3.52 *** [2.94, 4.22]
Mean Wind Speed (km/h)	0.94 ** [0.91, 0.98]	0.94 ** [0.91, 0.98]	0.94 ** [0.91, 0.98]	0.94 ** [0.91, 0.98]	0.94 ** [0.91, 0.98]	0.94 ** [0.91, 0.98]	0.94 ** [0.91, 0.98]
AIC	45,359	45,357	45,355	45,354	45,352	45,351	45,351

*** $p < 0.001$; ** $p < 0.01$; * $p < 0.05$.

Table S15 *Regression results from Generalized Linear Models for public housing spatial units (n =10,705), containing data from 2008 to 2020. Coefficients presented in this table have been exponentiated to obtain Incidence Rate Ratios (IRRs), for easier interpretation of results. Values in brackets represent 95% confidence intervals.*

Table S16: Regression Results of Private Housing Study Setting (2008-2020)

	(1)	(2)	(3)	(4)	(5)
(Intercept)	0.00 *** [0.00, 0.00]	0.00 *** [0.00, 0.00]	0.00 *** [0.00, 0.00]	0.00 *** [0.00, 0.00]	0.00 *** [0.00, 0.00]
Preceding Year Incidence Rate (Cases per 1,000 person-years)	1.01 *** [1.01, 1.02]	1.01 *** [1.01, 1.02]	1.01 *** [1.01, 1.02]	1.01 *** [1.01, 1.02]	1.01 *** [1.01, 1.02]
Year	1.10 *** [1.07, 1.13]	1.10 *** [1.07, 1.13]	1.10 *** [1.07, 1.13]	1.10 *** [1.07, 1.13]	1.10 *** [1.07, 1.13]
NDVI	0.26 * [0.08, 0.80]	0.26 * [0.08, 0.80]	0.28 * [0.09, 0.85]	0.29 * [0.10, 0.87]	0.30 * [0.10, 0.89]
Total Vegetation Area (%)	2.20 [0.17, 28.98]	2.59 [0.45, 14.84]	1.20 [0.46, 3.12]		
Forest Cover (%)	0.44 [0.02, 8.06]	0.37 [0.05, 2.92]			
Grass Cover (%)	71.04 * [2.46, 2053.33]	62.03 ** [3.05, 1261.06]	132.48 *** [9.06, 1936.25]	153.34 *** [12.47, 1885.89]	189.83 *** [15.49, 2326.08]
Managed Vegetation Cover (%)	1.31 [0.05, 31.77]				
Building Area (%)	4.48 ** [1.80, 11.14]	4.45 ** [1.80, 11.03]	4.68 *** [1.90, 11.53]	4.42 *** [1.85, 10.53]	4.43 *** [1.86, 10.56]

Number of Condominium Units	1.00 [0.99, 1.00]	1.00 [0.99, 1.00]	1.00 [0.99, 1.00]	1.00 [0.99, 1.00]	1.00 [0.99, 1.00]
Number of Landed Units	1.00 [1.00, 1.00]	1.00 [1.00, 1.00]	1.00 [1.00, 1.00]	1.00 [1.00, 1.00]	1.00 [1.00, 1.00]
Number of Public Housing Units	1.00 [1.00, 1.00]	1.00 [1.00, 1.00]	1.00 [1.00, 1.00]	1.00 [1.00, 1.00]	
Distance of Centroid to Drainage Network (m)	1.00 [1.00, 1.00]	1.00 [1.00, 1.00]	1.00 [1.00, 1.00]	1.00 [1.00, 1.00]	1.00 [1.00, 1.00]
Length of Drainage Network in Spatial Unit (m)	1.00 [1.00, 1.00]	1.00 [1.00, 1.00]	1.00 [1.00, 1.00]	1.00 [1.00, 1.00]	1.00 [1.00, 1.00]
Area Within 300m of a Waterbody (%)	1.89 *** [1.39, 2.57]	1.89 *** [1.39, 2.57]	1.94 *** [1.43, 2.63]	1.95 *** [1.44, 2.63]	1.97 *** [1.46, 2.67]
Area Within 500m of a Waterbody (%)	0.58 *** [0.45, 0.75]	0.58 *** [0.45, 0.75]	0.57 *** [0.44, 0.74]	0.57 *** [0.44, 0.74]	0.57 *** [0.44, 0.73]
Total Daily Rainfall (mm)	1.26 *** [1.18, 1.35]	1.26 *** [1.18, 1.35]	1.26 *** [1.18, 1.35]	1.26 *** [1.18, 1.35]	1.26 *** [1.18, 1.35]
Mean Temperature (°C)	5.08 *** [3.70, 6.97]	5.08 *** [3.70, 6.96]	5.05 *** [3.68, 6.92]	5.05 *** [3.68, 6.93]	5.10 *** [3.72, 7.00]
Mean Wind Speed (km/h)	1.31 *** [1.22, 1.41]	1.31 *** [1.22, 1.41]	1.32 *** [1.23, 1.42]	1.32 *** [1.22, 1.42]	1.31 *** [1.22, 1.41]
AIC	18,702	18,700	18,699	18,697	18,697

*** p < 0.001; ** p < 0.01; * p < 0.05.

Table S16 *Regression results from Generalized Linear Models for public housing spatial units (n =5,252), containing data from 2008 to 2020. Coefficients presented in this table have been exponentiated to obtain Incidence Rate Ratios (IRRs), for easier interpretation of results. Values in brackets represent 95% confidence intervals.*

References

Gaw, L. Y.-F., Yee, A. T. K., & Richards, D. R. (2019). A high-resolution map of singapore's terrestrial ecosystems. Data, 4(3), 116.

USGS. (2023a). Usgs. %5Curl%7Bhttps://www.usgs.gov/landsat-missions/landsat-normalized- difference-vegetation-index%7D

OneMap. (2023). Onemap api. %5Curl%7Bhttps://www.onemap.gov.sg/main/v2/%7D

Sun H, Dickens BL, Richards D, Ong J, Rajarethinam J, Hassim MEE, et al. Spatio-temporal analysis of the main dengue vector populations in Singapore. Parasit Vectors. 2021 Jan 11;14(1):41.

Kolimenakis, A., Heinz, S., Wilson, M. L., Winkler, V., Yakob, L., Michaelakis, A., Papachristos, D., Richardson, C., & Horstick, O. (2021). The role of urbanisation in the spread of aedes mosquitoes and the diseases they transmit—a systematic review. PLoS neglected tropical diseases, 15(9), e0009631.

Technical Assistance Document for the Reporting of Daily Air Quality- the Air Quality Index (AQI). Retrieved from https://www.airnow.gov/sites/default/files/2020-05/aqi-technical-assistance-document-sept2018.pdf

Fernandez, S. A., Sun, H., Dickens, B. L., Ng, L. C., Cook, A. R., & Lim, J. T. (2023). Features of the urban environment associated with aedes aegypti abundance in high-rise public apartments in singapore: An environmental case-control study. PLOS Neglected Tropical Diseases, 17(2), e0011075.

ECMWF. (2023). Resale flat prices. https://www.ecmwf.int/

DoS. (2023). Singapore department of statistics (dos). https://www.singstat.gov.sg/
